# Liver Injury by Carbon Tetrachloride Intoxication in 16 Patients Treated with Forced Ventilation to Accelerate Toxin Removal via the Lungs: A Clinical Report

**DOI:** 10.3390/toxics6020025

**Published:** 2018-04-27

**Authors:** Rolf Teschke

**Affiliations:** Department of Internal Medicine II, Division of Gastroenterology and Hepatology, Klinikum Hanau, 63450 Hanau, Academic Teaching Hospital of the Medical Faculty, Goethe University Frankfurt/Main, 60323 Frankfurt/Main, Germany; rolf.teschke@gmx.de

**Keywords:** carbon tetrachloride, aliphatic halogenated hydrocarbons, cytochrome P450 2E1, CO_2_-induced forced ventilation, hyperbaric oxygen treatment

## Abstract

Carbon tetrachloride (CCl_4_) is an efficient but highly toxic solvent, used in households and commercially in the industry under regulatory surveillance to ensure safety at the working place and to protect the workers’ health. However, acute unintentional or intentional intoxications by CCl_4_ may rarely occur and are potentially life-threatening. In this review article, therapy options are discussed that are based on a literature review of traditional poisoning cases and the clinical experience with 16 patients with acute poisoning by CCl_4_. Among various therapy options, the CO_2_-induced hyperventilation therapy will be considered in detail as the most promising approach. This special therapy was developed because only around 1% of the intoxicating CCl_4_ is responsible for the liver injury after conversion to toxic radicals via microsomal cytochrome P450 2E1 whereas 99% of the solvent will leave the body unchanged by exhalation. Therefore, to enhance CCl_4_ elimination through the lungs, CO_2_ is added to the inspiration air at a flow rate of 2–3 L min^−1^ in order to achieve hyperventilation with a respiratory volume of 25–30 L min^−1^. Under this therapy, the clinical course was favorable in 15/16 patients, corresponding to 93.8%. In essence, patients with acute CCl_4_ intoxication should be treated by forced ventilation.

## 1. Introduction

Carbon tetrachloride (CCl_4_) is known for its hepatotoxic potency and was previously often used as effective solvent and cleaning agent in industrial manufactories, households, dry-cleaning textile laundries, in fire extinguishers, as a precursor of refrigerants or rocket propellant, and also appreciated in humans as an effective anthelmintic chemical for treating ankylostomiasis [1,2,3,4,5,6]. Ingested as a chemical drug to treat helminths diseases, CCl_4_ had a long tradition and was classified as a typical drug with reference to more than 30,000 treatments in humans without marked symptoms at doses of 1–4 mL as described in 1923 [1]. Based on its chemical structure, CCl_4_ is a typical halogenated or more specifically a chlorinated hydrocarbon molecule also called tetrachloromethan, because all 4 hydrogen atoms of methane are substituted by chloride atoms closely tied with the single C atom in the center [2,3,4,5,6]. These are perfect conditions for an efficient solvent that keeps other chemical products in solution without reacting with these. CCl_4_ is continuously used in experimental models of reproducible liver injury which explains the large number of scientific reports on studies of liver injury caused by this chemical [2,3,4,5,6,7,8]. Additional information on the cascade of events leading to CCl_4_-mediated acute liver injury was also provided by experiments comparing liver enzyme activities such as GDH (glutamate dehydrogenase), ALP (alkaline phosphatase), ALT (alanine aminotransferase), or AST (aspartate aminotransferase), in the serum with levels of CCl_4_ in blood, liver and fat, using the rapid head space method of gas chromatography (GC) [8], with details described in earlier publications [9,10]. 

Liver injury observed in CCl_4_ poisoning is due to toxic metabolites of CCl_4_ rather than to the parent chemical [2,3,4,5,6,7,11], catalyzed by the hepatic microsomal cytochrome P450 (CYP), especially by its isoenzyme CYP2E1 [5,6] that is also a component of the hepatic microsomal ethanol-oxidizing system (MEOS) involved in the hepatic metabolism of ethanol [12,13,14]. The CYP2E1 content and MEOS activity are inducible following prolonged alcohol use [12], and even a single dose of ethanol is sufficient to induce MEOS activity [15]. Triggered by CYP2E1, chronic alcohol consumption predisposes to experimental liver injury by CCl_4_ [11] and explains acute hepatic interactions between alcohol and CCl_4_ [16]. In patients acutely intoxicated by CCl_4_, alcohol is a major issue whether ingested concomitantly or used chronically before. 

Understanding CCl_4_-related molecular mechanisms is prerequisite to efficiently treat patients acutely intoxicated from CCl_4_ and to develop new therapy options. Because most of the CCl_4_ taken up by the body will be eliminated by expiration through the lungs, a therapy of forced ventilation had been developed to enhance toxin removal. Additional efforts must be directed to reduce microsomal production of toxic metabolites derived from CCl_4_ by searching for appropriate inhibitors of microsomal drug metabolizing enzymes, in addition to cimetidine that is presently used for this purpose. 

This article provides an overview on recent developments of treatment modalities for patients with acute intoxications by carbon tetrachloride and discusses the use of the CO_2_-induced hyperventilation in 16 patients. These present cases were compared to historical cases published from time to time since 1922. 

## 2. Literature Search

A computerized search of the Medline database was used with the following two search terms: human acute carbon tetrachloride intoxication case reports, providing around 831,000 hits, and human acute carbon tetrachloride liver injury case reports, providing around 654,000 hits. The yield of assessable cases was poor, due to interfering experimental reports and limited use of CCl_4_, being largely been removed from the market, replaced by other less toxic solvents, or used under regulatory occupational restrictions.

## 3. Historical Cases of CCl_4_ Poisoning and Liver Injury Caused by CCl_4_

Clinical features of human CCl_4_-induced liver injury and CCl_4_ poisoning in general have been well described in a variety of publications [17,18,19,20,21,22,23,24,25,26,27,28,29,30,31,32,33]. Most of these clinical details described are expected as transferable from results obtained in animal studies [2,3,4,5,6]. The impact on human toxicity was well assessable only with the development of analytical methods and its introduction in clinical practice. For instance, the valid detection of liver injury by blood analysis was established at a time when serum activities of transaminases such as AST or ALT could be measured. Reviewing the traditional cases of patients with CCl_4_ intoxication provides a good clinical insight in this toxic disease. Originally used as an anthelminthic drug without knowledge of its hepatotoxic and nephrotoxic properties, CCl_4_ was then appreciated for many purposes as an effective solvent in household and industry but it was mostly used without any safety measures to protect human health because CCl_4_ was viewed as harmless chemical. Indeed, one of the earliest, still vague description of liver injury goes back to a publication in the second volume of the BMJ in 1922 [17], followed by CCl_4_ poisoning in an occupational context [18]. Surprisingly, historical reports provided little new ideas how to accelerate CCl_4_ removal out of the intoxicated body, nor were there any significant suggestions how to decrease the high lethality rate associated with acute CCl_4_ intoxications, except perhaps considering hemodialysis to treat renal failure in severe intoxications. Details of selected historical cases are presented for a broad overview (Table 1).

Some results of the historical reports are of major clinical interest or remain an actual issue (Table 1). For instance, suggestions for treatment included hyperbaric oxygen therapy, hypothermia, oral use of calcium lactate, a diet high in carbohydrates, intravenous administration of dextrose and calcium gluconate, rectal application of dextrose, and the use of *N*-acetylcysteine. Only a few of these therapy options are based on experimental studies, rarely verified in patients with CCl_4_ intoxication. Key points of the historical cases are presented in condensed form for a quick clinical overview (Table 2).

## 4. Actual Patients with CCl_4_ Poisoning and CO_2_-Induced Hyperventilation

As opposed to historical reports on previous patients, who did not receive a specific therapy directed against CCl_4_ as the poisonous chemical (Table 1 and Table 2) [17,18,19,20,21,22,23,24,25,26,27,28,29,30,31,32,33], some actual recommendations how to diagnose and treat patients intoxicated by acute ingestion or inhalation of CCl_4_ or other aliphatic halogenated hydrocarbons have been presented in a recent report [34]. Essential diagnostic requirements at admission and during the clinical course are now outlined in a tabular listing (Table 3), which should be followed in a setting of an intensive care unit and at best within a center with special experience with these intoxications as described [34]. The diagnostic workup of the patient includes among many other essentials also the determination of CCl_4_ in the blood by GC [10] or in the exhalation air by the Draegerunknown-tube^®^ system (DTS) supplied by Draeger, Lübeck in Germany [34,35]. Details of using the DTS are provided online [35] and in the legend of Table 3. In addition to diagnostic recommendations for clinical practice in acute CCl_4_ intoxication (Table 3), special attention is placed on therapy aspects with focus on forced ventilation achieved as CO_2_-induced hyperventilation to accelerate CCl_4_ removal via the lungs (Table 4 and Table 5) [34], supplemented by data on the acid-base balance essential for clinical treatment (Table 6). These recommendations result from previous experience as outlined in various publications [10,36,37,38,39,40,41,42]. 

The hyperventilation therapy was applied in 16 patients with acute CCl_4_ intoxication, their case narratives help characterize the specific toxic disease caused by CCl_4_ and are presented including some selected clinical details (Table 7). Among the overall 16 patients with acute CCl_4_ poisoning, for 13 patients CCl_4_ was the only poison confronting the patients whereas in 3 other patients CCl_4_ was causative as mixture with other aliphatic halogenated hydrocarbons. Apart from a few younger patients, most of the 16 patients were adults with an age of up to 70 years. The male: female ratio was 10:6. Intoxication occurred via inhalation (*n* = 7) or ingestion (*n* = 9), by intention (*n* = 10) or unintentionally (*n* = 6). The duration of the hyperventilation therapy was variable in the 13 patients who had been intoxicated by CCl_4_ alone, with a longer treatment in those with an intentional intoxication as compared to those who were intoxicated unintentionally (156.0 ± 32.7 h vs. 87.0 ± 13.4 h; *p* < 0.01). For most patients who ingested CCl_4_, the approximate amount of the swallowed toxin could fairly well be documented. However, only part of the ingested CCl_4_ will exert its toxic property in the body because vomiting was a common feature in many patients and gastro-intestinal lavage likely contributes to reduce the quantity of CCl_4_ in the body. 

**Figure 1:**
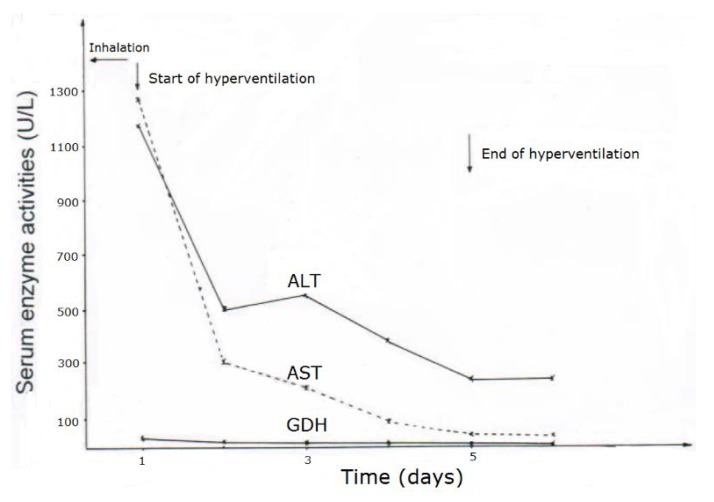
Patient 11 intoxicated by inhalation of CCl_4_ (unknown amounts), presenting serum activities of ALT, AST, and GDH under CO_2_-induced hyperventilation therapy. Abbreviations: ALT, Alanine aminotransferase; AST, Aspartate aminotransferase; GDH, Glutamate dehydrogenase.

**Figure 2:**
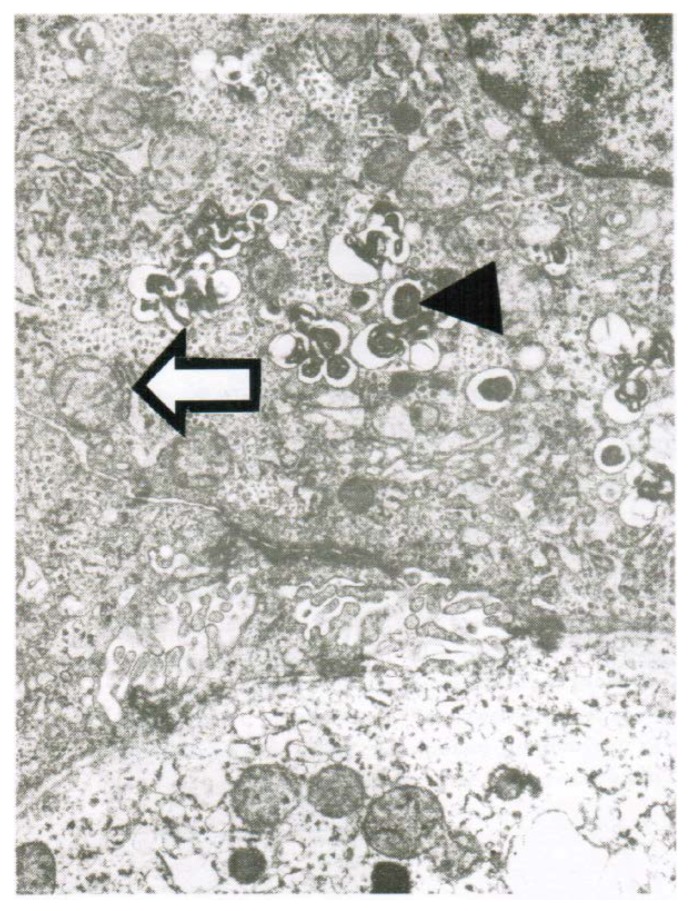
Patient 11 with intoxication of CCl_4_ by inhalation (unknown amounts): Liver tissue specimen for electron microscopy (18,500-fold magnification) was obtained on day 5 after admission. In addition to abundant bile pigments (◄), as sign of major subcellular injury liver mitochondria are slightly swollen and their cristae are reduced (**⇦**).

**Figure 3:**
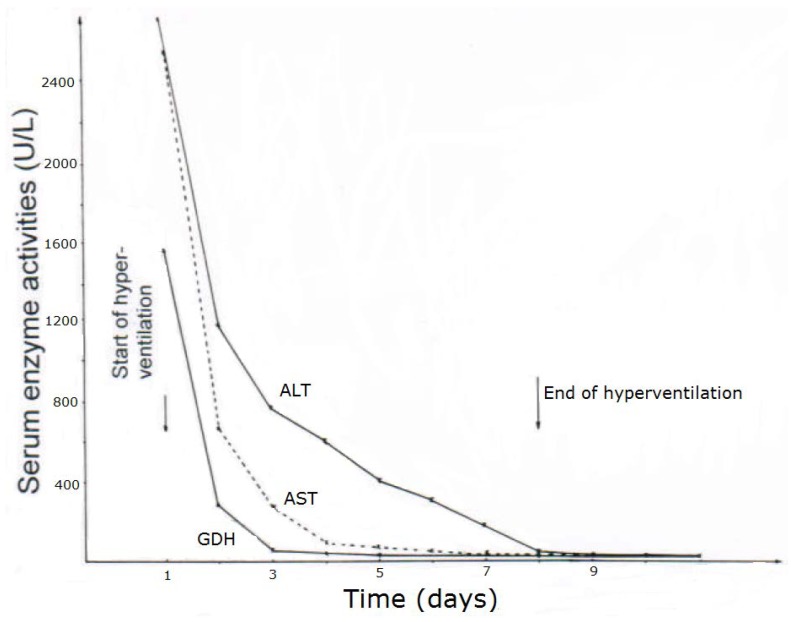
Patient 12 with CCl_4_ intoxication by inhalation (unknown amounts). Serum activities of ALT, AST, and GDH after intoxication and during CO_2_-induced hyperventilation therapy. Abbreviations: ALT, Alanine aminotransferase; AST, Aspartate aminotransferase; GDH, Glutamate dehydrogenase.

**Figure 4:**
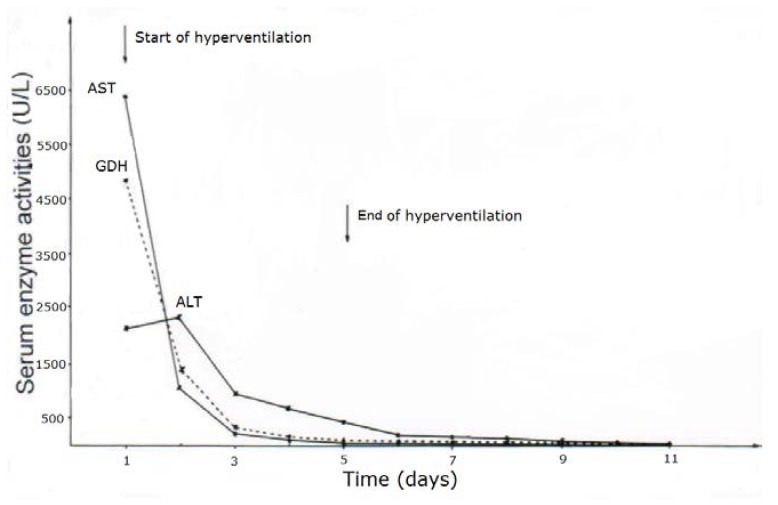
Patient 13 with CCl_4_ intoxication by inhalation (unknown amounts) and serum activities of AST, ALT, and GDH following poisoning and under CO_2_-induced hyperventilation therapy. Abbreviations: ALT, Alanine aminotransferase; AST, Aspartate aminotransferase; GDH, Glutamate dehydrogenase.

CCl_4_ may cause abnormal liver tests (LTs) at a variable extent as evidenced by increased serum activities of AST, ALT, and GDH, but time of peak occurrence depends on the route and duration of toxin uptake (Table 7). Facilitating rapid toxin absorption through the bronchial mucosa, inhalation of CCl_4_ at intoxicating amounts leads to variably increased serum activities of AST, ALT, and GDH found already at admission in 3 patients with a subsequent rapid decline (Table 7, cases 11–13). In one of the 3 patients (case 11), AST was higher than ALT, but GDH was only little increased at hospital admission (Figure 1). Interestingly, electron microscopy of a liver tissue specimen obtained on day 5 after admission and at the day when hyperventilation has been discontinued (Figure 1) revealed toxic injury of mitochondria that appeared swollen associated with a reduction of their cristae, and deposits of bile pigments (Figure 2). In the 2 other patients intoxicated by CCl_4_ inhalation (cases 12 and 13) (Table 7), the serum activity of AST was higher compared to ALT (Figure 3 and Figure 4), but GDH activities were extremely high with 1534 U/L in case 12 (Figure 3) and with 4746 U/L in case 13 (Figure 4). In the latter patient, this high GDH activity reflects severe toxicity towards the liver and is in line with severe renal toxicity with serum creatinine values of 14.5 mg/dL on day 5 and subsequent requirement of hemodialysis due to renal failure (Table 7, case 13). It seems that the first hit after CCl_4_ intoxication is directed to the liver followed by the kidneys.

**Figure 5:**
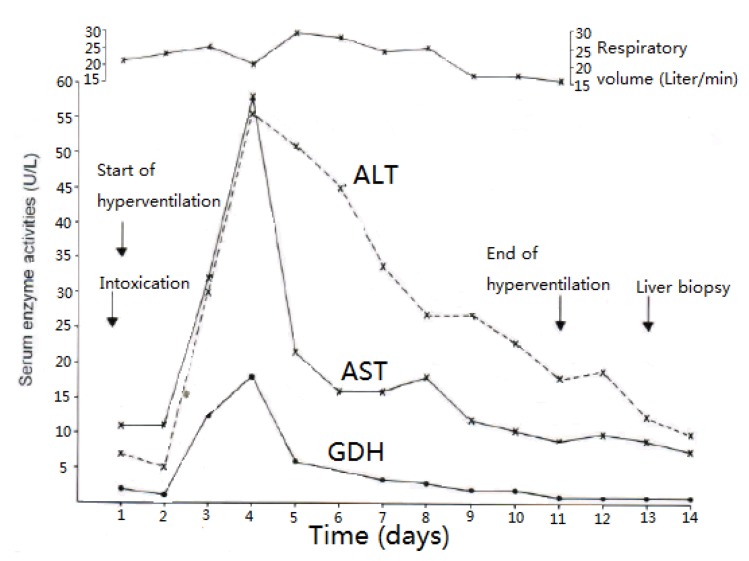
Patient 1 after ingestion 30 mL CCl_4_ and serum activities of AST, ALT, and GDH under CO_2_-induced hyperventilation therapy. Abbreviations: ALT, Alanine aminotransferase; AST, Aspartate aminotransferase; GDH, Glutamate dehydrogenase.

**Figure 6:**
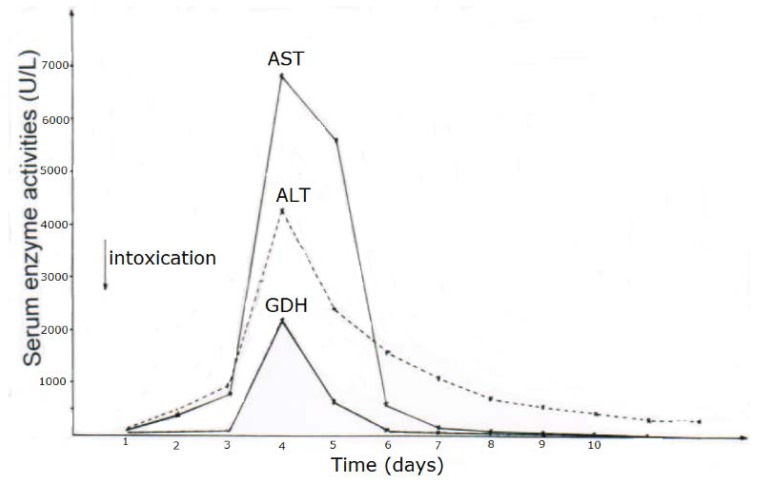
Patient 5 with ingestion of 100 mL CCl_4_ and serum activities of AST, ALT, and GDH during CO_2_-induced hyperventilation therapy for 10 days. Abbreviations: ALT, Alanine aminotransferase; AST, Aspartate aminotransferase; GDH, Glutamate dehydrogenase.

As opposed to CCl_4_ intoxication by inhalation, case analyses showed that acute ingestion of CCl_4_ with its delayed gastrointestinal absorption results in abnormal LTs only during the hospital stay and therefore usually between days 4 and 5 after intoxication, shown as example for 2 patients (Table 7, cases 1 and 5), with details presented for patient 1 (Figure 5) and patient 5 (Figure 6). In many cases, there is only a minimal or moderate increase of LTs that is associated with normal values of total bilirubin, a parameter of liver function as opposed to AST and ALT reflecting LTs and therefore diagnostic parameters of liver injury but not those of liver function (Table 7). Following intoxications with high amounts of ingested CCl_4_ in rare cases, total bilirubin starts to increase at the day after ingestion with possibly high values in the further course together with clinical features of jaundice, signifying severe disturbances of liver functions. Except for severe intoxications where serum creatinine values start to increase on day 2 after intoxication, enhanced serum levels of creatinine or emerging renal failure were rarely observed (Table 7), likely as a consequence of daily creatinine measurements, assessing fluid balance, and early initiation of forced diuresis using intravenous electrolytes and furosemide in line with recommendations (Table 4). 

**Figure 7:**
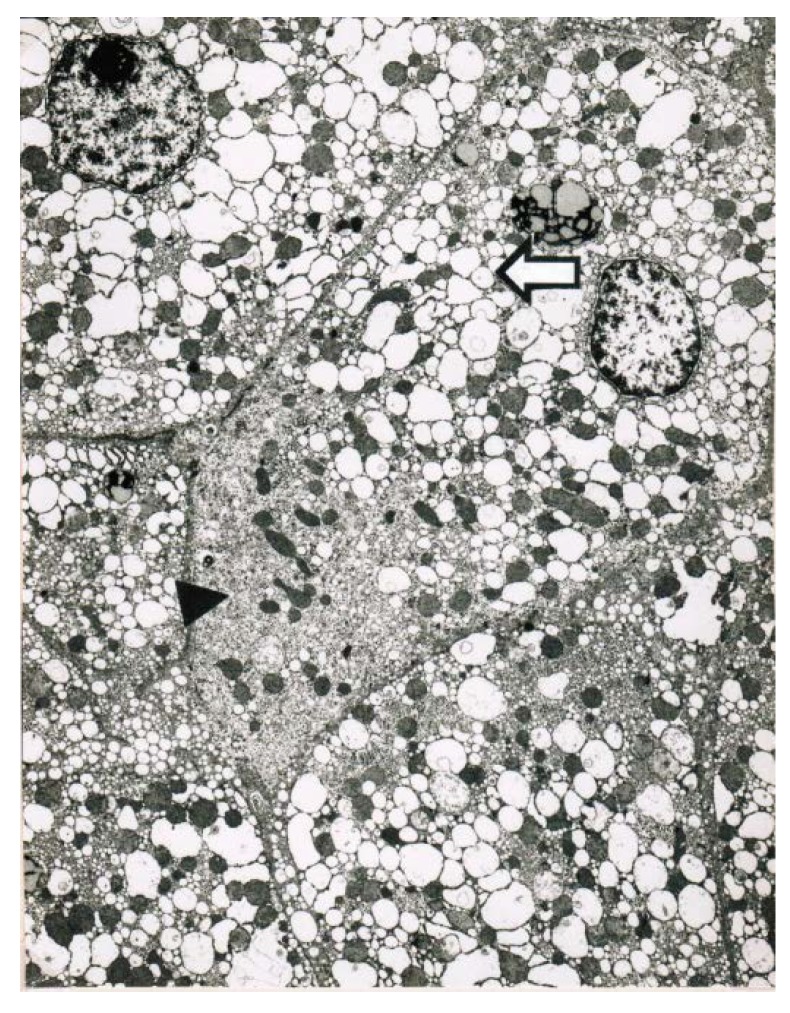
Patient 5 with poisoning by CCl_4_ ingestion (50 mL) and liver tissue specimen obtained on day 14 after intoxication for electron microscopy (5200-fold magnification). Key features include a striking proliferation of the smooth endoplasmic reticulum of the hepatocyte (►) with close by injured mitochondria, and in addition to a pronounced dilatation of the smooth endoplasmic reticulum presenting as dilated cisterns (**⇦**).

**Figure 8:**
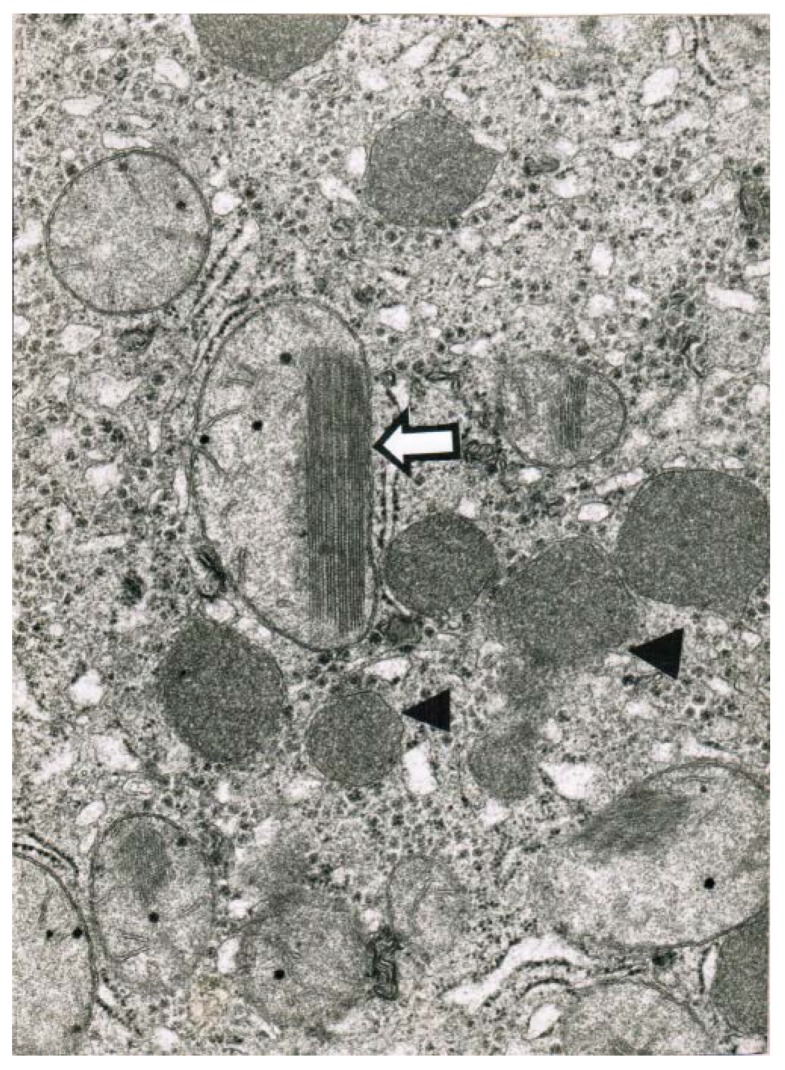
Patient 2 with CCl_4_ ingestion (10–20 mL) showed 4 days after termination of the CO_2_-induced hyperventilation therapy by electron microscopy (32,000-fold magnification) a reduction and disorganization of hepatic mitochondria, some of which contained crystalline inclusion bodies. A mega-mitochondria revealed loss of cristae and a pronounced crystalline inclusion (**⇦**). In other parts there is an increase of lysosomes (◄).

Obtained shortly after cessation of the forced ventilation therapy and at times of normal or near normal serum AST and ALT activities, liver histology was devoid of severe confluent liver cell necroses but showed occasionally moderate steatosis and signs of remnant liver injury such as inflammatory cells or rarely single liver cell necrosis (Table 7). However, at the same time electron microscopy data still indicate severe liver injury especially related to mitochondria, shown for 2 patients (Figure 7 and Figure 8) with case narratives presented earlier (Table 7, cases 2 and 5). Several weeks or months after discharge patients and their doctors have been contacted to assess the further clinical course. Data provided for some patients indicated that the CCl_4_-liver injury was self-limited without major health of liver problems. However, in severe intoxications the risk of a life-threatening course will remain if a point of no return is achieved.

Regrettably, the clinical course was lethal for one patient (Table 7, case 9) among the 16 patients (Table 7), which corresponds to a lethality rate of 6.3%. In this patient (case 9), liver transplantation was considered but declined due to contraindications including pneumonia, multi-organ failure and still detectable CCl_4_ in the blood. Although information on the patients with CCl_4_ intoxication is available, the present data are limited and do not allow defining the lethal dose of CCl_4_ in the cohort of assessed patients due to confounding variables of vomiting, gastro-intestinal lavage, and forced ventilation, which differently contribute to toxin removal. 

## 5. Clinical and Experimental Challenges of CO_2_-Induced Hyperventilation 

### 5.1. Clinical Data 

CO_2_-induced hyperventilation therapy was initially suggested as a therapy option for intoxications by trichloroethylene, a commonly used aliphatic halogenated hydrocarbon in France, and clinical and experimental evidence of efficacy was concomitantly provided [37]. This new therapy aimed to accelerate the elimination of volatile solvents through increased exhalation and created interest in Germany at hospitals of the Heinrich Heine University in Düsseldorf [38,39], first applied in 110 children to treat intoxications by various aliphatic hydrocarbons as summarized in 1979 [39] and later also used in adults with initial results on 13 patients published in 1977 [38]. Details of the historical background on the experience in Düsseldorf have been summarized recently [34], with related clinical and experimental data provided in additional publications [8,9,10,16,36,40,41,42,43,44,45].

Based on the initial study of F. Pebay-Peyroula and A. M. Nicaise published in 1970 [37], it became increasingly more clear that in future patients with acute intoxications by aliphatic halogenated hydrocarbons should be treated by CO_2_-induced hyperventilation because additional supporting evidence was provided that this approach can enhance toxin removal via the lungs in patients [10,37,38,39]. Under these conditions it appeared unethical to conduct a randomized clinical trial (RCT), similar to other treatment conditions with the purpose to evaluate efficacy. Any RCT would require homogeneity of 2 cohorts, one receiving the CO_2_-induced hyperventilation therapy and the other one receiving some kind of a control therapy, hardly to be defined and open for future discussions. Cohort homogeneity is poorly achievable and uncertain, because the causative hydrocarbons vary from one patient to the other, and variability expands to the amount ingested or inhaled. Confounding variables include acute or previous chronic alcohol use and as to whether the patients did experience vomiting, decreasing the amount of toxins that could be injurious. Consequently, there have been no good arguments to conduct such RCT to verify efficacy. Instead, additional studies were carried to systematically evaluate best conditions applying the CO_2_-induced hyperventilation and to establish the impact of this therapy on toxin elimination.

**Figure 9:**
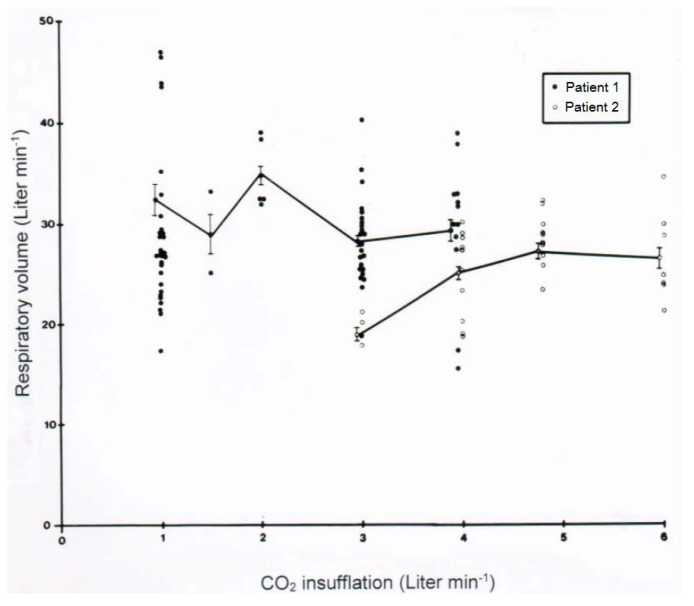
Effect of a variable CO_2_ flow min^−1^ on the respiratory volume min^−1^ was assessed in 2 patients.

Of clinical interest are data on the acid-base balance analyzed under clinical CO_2_-induced hyperventilation in 5 patients who had ingested CCl_4_ in various amounts (Table 6). The results show some variability among the assessed patients and ask for continuous re-analysis when this therapy is used. In another set of a clinical study, the effect of a variable CO_2_ flow min^−1^ on the respiratory volume min^−1^ was assessed in 2 patients (Figure 9). One of the patients appears as a good responder by reaching a respiratory volume of 25–30 L min^−1^ or slightly above with a CO_2_ flow of 1.5–4.0 L min^−1^. In the other patient and rarely in additional patients, the response is poor, because the desired respiratory volume was not achieved with CO_2_ at a flow rate of 3 L min^−1^ but required a somewhat higher flow rate of 4–5 L min^−1^. However, increasing the flow rate above 5 L min^−1^ is unsuccessful (Figure 9) and not recommended. Consequently, in all patients the initial flow rate of CO_2_ should be 2–3 L min^−1^, to be adjusted and titrated according the obtained respiratory minute volume. 

**Figure 10:**
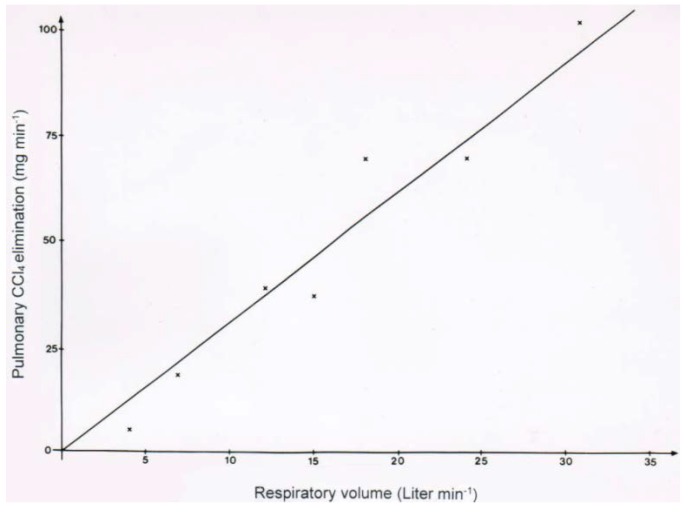
In a patient intoxicated by CCl_4_ ingestion and treated with CO_2_-induced hyperventilation, CCl_4_ disposal via the lungs was quantitatively assessed in relation to the respiratory minute volume, showing that the amount of CCl_4_ eliminated is dependent on the respiratory minute volume achieved.

With a newly described rapid analytical method using the head space technique of GC, a quick quantitative determination of CCl_4_ in the blood of patients intoxicated by CCl_4_ became feasible and allowed an improved clinical management of these patients [10]. For instance, in a patient intoxicated by ingested CCl_4_ and treated with CO_2_-induced hyperventilation, CCl_4_ disposal via the lungs was quantitatively assessed in relation to the respiratory minute volume (Figure 10). The results clearly show that the amount of CCl_4_ eliminated is dependent on the respiratory minute volume achieved.

**Figure 11:**
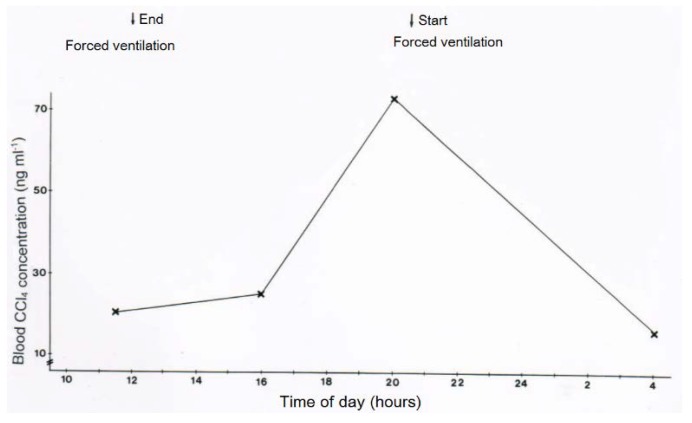
In a patient with CCl_4_ intoxication by ingestion, blood levels of CCl_4_ were determined with or without forced ventilation induced by CO_2_. Blood CCl_4_ levels increased when forced ventilation was terminated and decreased again with reinstitution of forced ventilation.

**Figure 12:**
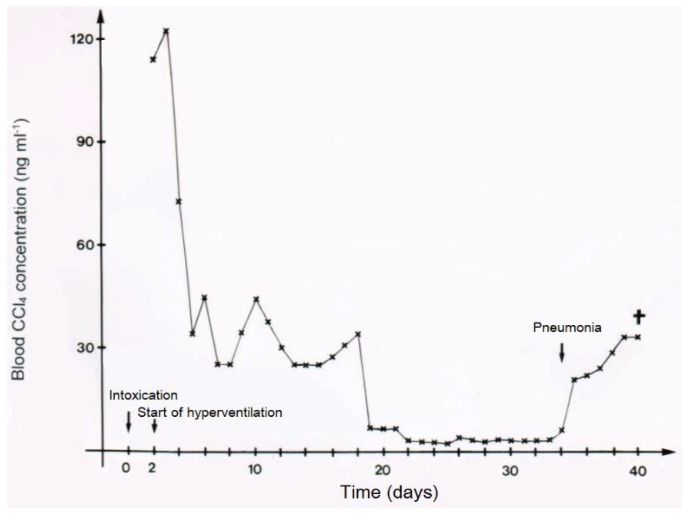
In another patient (case 9) intoxicated by oral use of 50 mL CCl_4_, blood levels of CCl_4_ declined under the hyperventilation therapy and reached a plateau that lasted for around 16 days before a striking increase of blood levels was observed, due to impaired CCl_4_ elimination via the lungs as a consequence of an emerging pneumonia with fatal outcome, reproduced with permission from [10]. Copyright Springer, 1983.

In two patients with CCl_4_ intoxication by ingestion, blood levels of CCl_4_ were determined under various ventilation conditions (Figure 11 and Figure 12). Intermittent discontinuation of the CO_2_-induced hyperventilation led to a striking increase of CCl_4_ levels in the blood, whereas its re-introduction reduces CCl_4_ to levels achieved under the previous hyperventilation regimen (Figure 11). In another patient intoxicated by oral use of CCl_4_, blood levels of CCl_4_ declined under the hyperventilation therapy and reached a plateau that lasted for around 16 days before a striking increase of blood levels was observed, due to an impaired CCl_4_ elimination via the lungs as a consequence of an emerging pneumonia with fatal outcome (Figure 12). 

Follow-up data were provided by physicians or patients for only 5/12 patients following acute CCl_4_ intoxication by ingestion or inhalation. Requests had been confined to serum activities of ALT, AST, and GGT, all of which remained in the normal range or normalized within 7–10 days after discharge, considering 3 patients who had already normal values at discharge, and the two other patients with slightly increased values at discharge. In all five patients, all values remained in the normal range also for 3 to 7 years after intoxication. Based on the results of this small cohort, there is no evidence of long-term hepatic sequelae, such as vanishing bile duct syndrome, in patients who experienced acute CCl_4_ intoxications.

Clinical evidence suggests the efficacy of CO_2_-induced hyperventilation by accelerating CCl_4_ removal via the lungs (Figure 9, Figure 10, Figure 11 and Figure 12). However, these data do not allow the firm conclusion that this therapy reduces the overall lethality rate in patients with CCl_4_ intoxication [36], which was estimated at 28–35% until 1953 and at 17% thereafter until 1965 due to increased use of dialysis devices [25], but with 25% this rate was somewhat higher in a study from Düsseldorf published in 1969 without applying the new CO_2_-induced hyperventilation therapy [25]. These figures compare with the lethality rate of 6.7% observed with 16 patients treated with forced ventilation (Table 7), but such comparisons of various clinical cohorts with their case variabilities are uncertain and open for discussion. Another approach using studies in animal models provides additional supporting data.

### 5.2. Experimental Results

**Figure 13:**
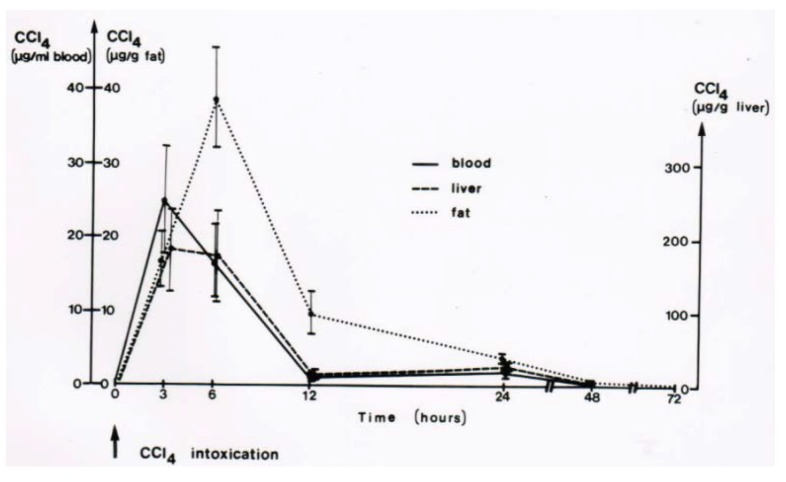
In this rat model, CCl_4_ administered by gavage is found within 3 h in the liver and the blood, and with higher CCl_4_ amounts in the fat around 6 h after gavage. CCl_4_ levels then decline, more quickly in the blood and the liver as compared to the fat. Figure reproduced with permission of the publisher from a previous report [8]. Copyright Elsevier, 1983.

CO_2_-induced hyperventilation is an essential part of the overall new therapeutic approach for acute poisonings by CCl_4_ in humans (Table 3, Table 4, Table 5 and Table 6), and animal studies are suggestive of its efficacy [43,44,45]. First of all, basic knowledge is essential on the distribution of orally applied CCl_4_ in animals that mimic conditions of intoxicated humans (Figure 13) [8]. In this rat model, CCl_4_ administered by gavage is found within 3 h in the liver and the blood, and with higher CCl_4_ amounts in the fat around 6 h after gavage. CCl_4_ levels then decline, more quickly in the blood and the liver as compared to the fat (Figure 13). In other studies comparing the time course of CCl_4_ levels in the blood with serum activities of liver enzymes, peak levels of CCl_4_ are found at 3 h after gavage and activity peaks of ALT and AST then between 12 and 24 h after gavage, followed by GDH and ALP with a peak at 48 and 72 h [8]. Compared to CCl_4_ poisonings by ingestion in humans (Table 7, Figure 5 and Figure 6), changes of serum enzyme activities occur earlier in the animals [8]. The CCl_4_-induced rise of serum ALT activity is associated with a corresponding decline of ALT activity in the liver [8], indicating that the liver is likely the origin of the increased ALT activity in the serum. Under these experimental conditions, other enzyme activities in the liver are also reduced [8], again likely due to an increased efflux out of the liver into the blood. 

**Figure 14:**
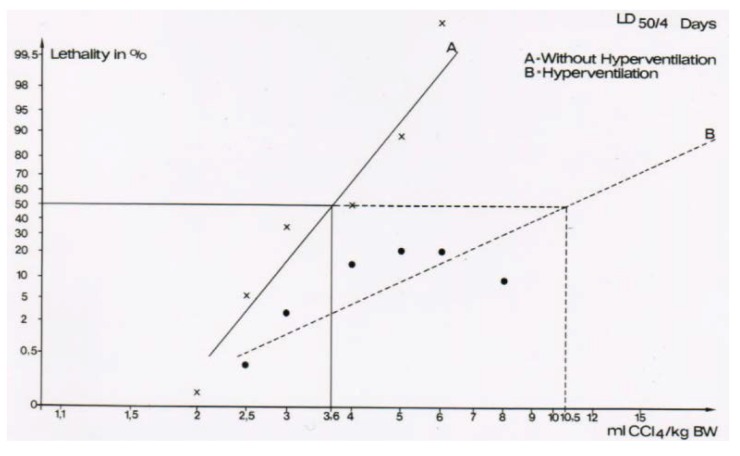
The lethal dose (LD) was determined as LD_50_ for 4 days, with 3.6 ± 0.5 mL CCl_4_ per kg body weight for the non-hyperventilated animals versus 10.5 ± 3.0 mL CCl_4_ per kg body weight for the hyperventilated animals. The difference was statistically significant using the chi-square test (*p* = 0.015), reproduced with permission from [44,45]. Copyright Wiley, 1982 and Springer, 1982.

**Figure 15:**
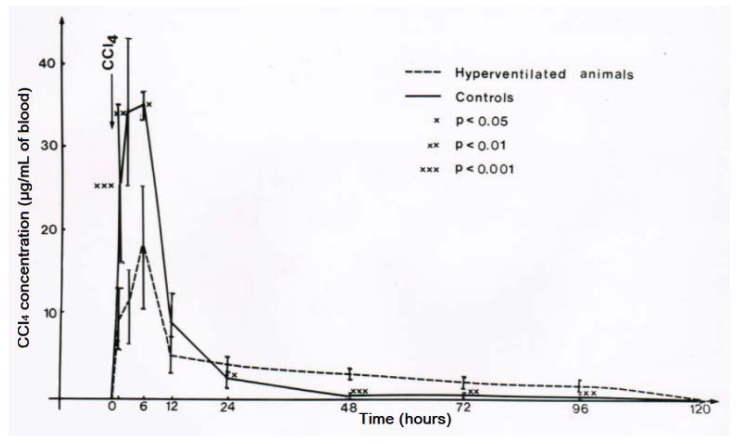
Experimental hyperventilation leads to a reduction of CCl_4_ levels in the blood, reproduced with permission from [43]. Copyright Springer, 1983.

**Figure 16:**
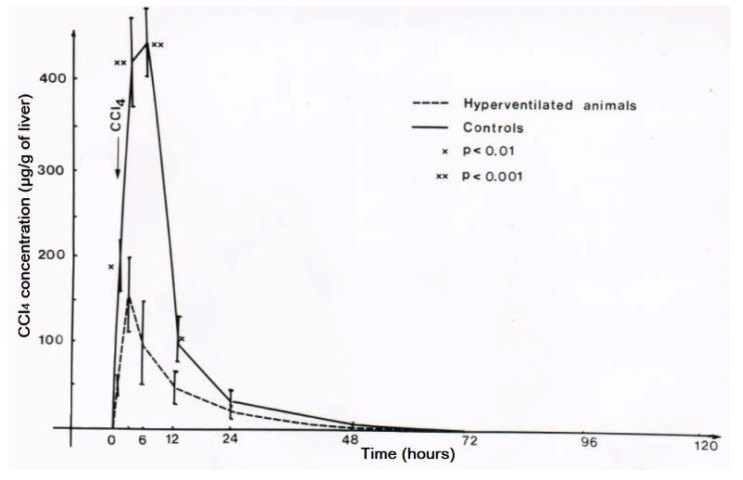
Experimental hyperventilation reduces CCl_4_ levels in the liver, reproduced with permission from [43]. Copyright Springer, 1983.

**Figure 17:**
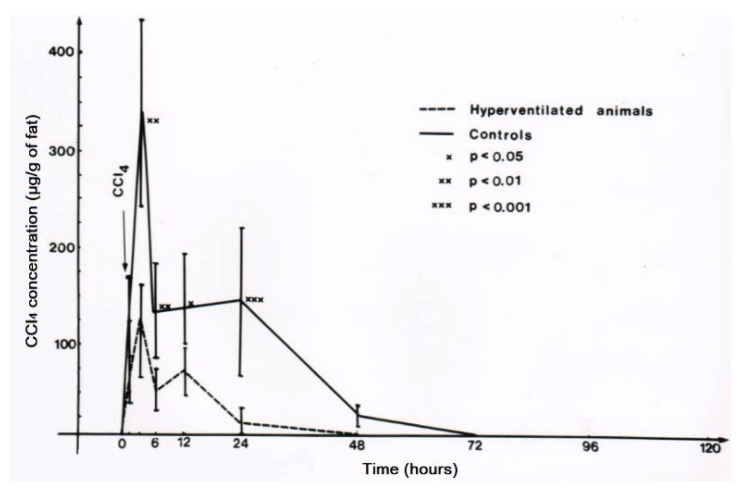
Experimental hyperventilation reduces CCl_4_ in the fat tissue where it is soluble and can be quantified, reproduced with permission from [43]. Copyright Springer, 1983.

For experimental hyperventilation and the question of efficacy, female rats received 2.5 mL CCl_4_ per kg body weight as mixture with olive oil (1:1) and applied by gavage. Half of the animals were placed in a chamber ventilated by air in which part of the nitrogen was substituted by CO_2_ that caused an increase of the respiratory frequency by 50%. The other half of the CCl_4_-treated animals were kept in another chamber and had access to a similar gas mixture that did not contain CO_2_ [43,44]. The lethal dose (LD) was determined as LD_50_ for 4 days, with 3.6 ± 0.5 mL CCl_4_ per kg body weight for the non-hyperventilated animals versus 10.5 ± 3.0 mL CCl_4_ per kg body weight for the hyperventilated animals (Figure 14) [44,45]. The difference was statistically significant using the chi-square test (*p* = 0.015). These results led to the conclusion that experimental CO_2_-induced hyperventilation is effective in reducing short-term lethality due to acute CCl_4_ intoxication by gavage [44]. Of interest are other comparative studies of experimental CO_2_-induced hyperventilation versus lacking hyperventilation on CCl_4_ levels [43]. These levels are all reduced under conditions of experimental hyperventilation, not only in the blood (Figure 15), but also in the liver (Figure 16), and the fat (Figure 17) [43]. Experimental CO_2_-induced hyperventilation also ameliorated liver injury by CCl_4_ as assessed by liver histology [44]. In comparison to non-hyperventilated animals showing pronounced centrilobular necroses and signs of steatosis, hyperventilated animals presented only few signs of liver injury. In addition, experimental CO_2_-induced hyperventilation partially prevented the increase in serum activities of AST, ALT, and GDH [43,44]. These experimental data substantiate the beneficial effect of CO_2_-induced hyperventilation on CCl_4_-induced liver injury and related lethality.

## 6. CCl_4_ and Hepatic Microsomal CYP 2E1 

### 6.1. Carbon Tetrachloride

CCl_4_ is soluble in fat where it can be quantified (Figure 13 and Figure 17) [8,43], but with 0.08 g/100 mL water it is virtually insoluble in water [41,46]. For clinical purposes it is important that renal CCl_4_ excretion per hour is low with <0.6% of the dose taken up, conditions not recommending forced diuresis for increasing renal CCl_4_ excretion although this approach early applied is nephroprotective [41]. Conversely, total CCl_4_ excretion in breath after 1 h is as much as 33% of the dose taken up [40,46], and this is why in CCl_4_ intoxication forced ventilation is recommended to accelerate CCl_4_ removal via the lungs. 

**Figure 18:**
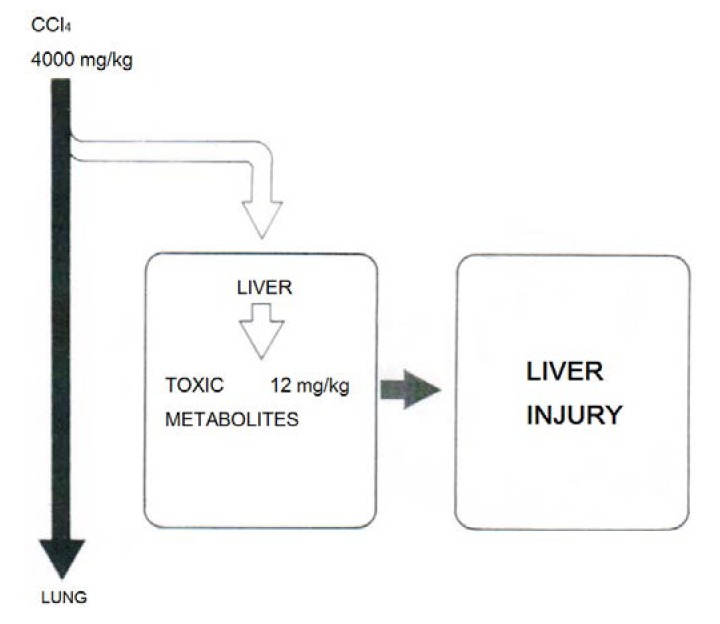
Experimental data suggest that only around 1% of the incorporated CCl_4_ is responsible for liver injury while 99% thereof will leave the body unchanged via the lungs, reproduced with permission from [47]. Copyright Schattauer, 1975.

Agreement exists that only around 1% of the incorporated CCl_4_ is responsible for liver injury while 99% thereof will leave the body unchanged via the lungs (Figure 18) [29,47]. This requires additional efforts to minimize toxic effects by CCl_4_ and its metabolites at the microsomal level.

### 6.2. Cytochrome P450 2E1 

**Figure 19:**
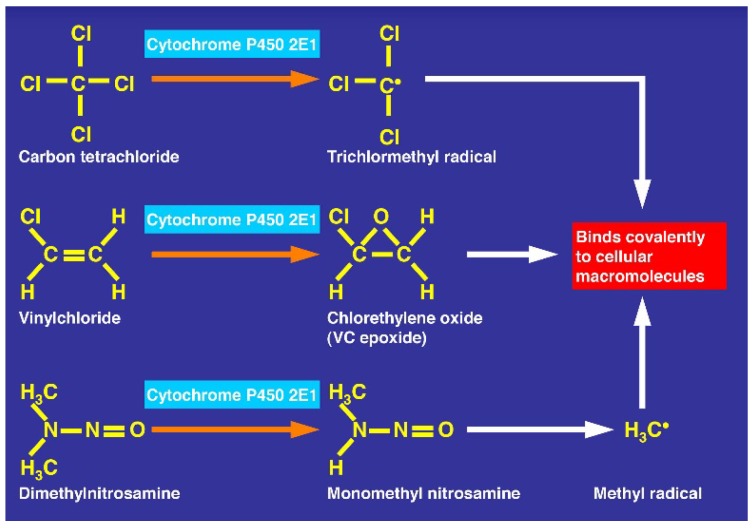
CCl_4_ metabolized by cytochrome P450 2E1 similarly to other toxins such as vinyl chloride and dimethylnitrosamine.

**Figure 20:**
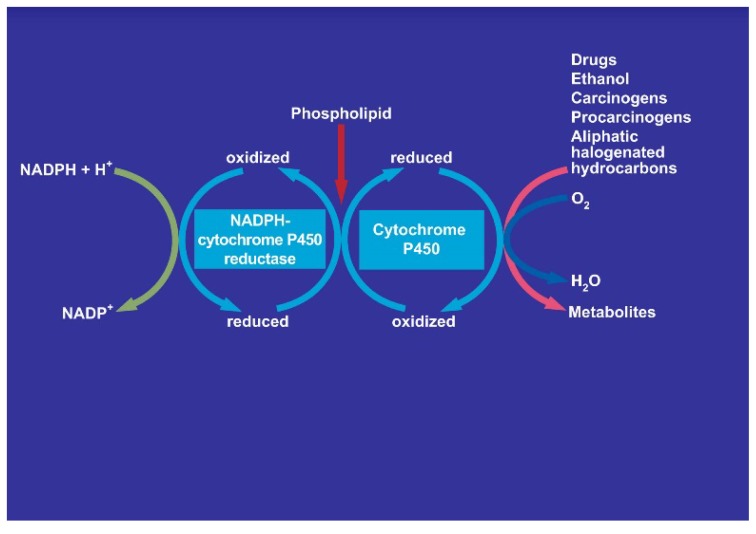
Involvement of cytochrome P450 in the microsomal metabolism of various substrates including aliphatic halogenated hydrocarbons with carbon tetrachloride as example. The NADPH-cytochrome P450 uses NADPH + H^+^ and will itself be reduced, allowing the cytochrome P450 to be transferred from the oxidized state to the reduced state. The overall reactions needs also molecular oxygen and phospholipids.

**Figure 21:**
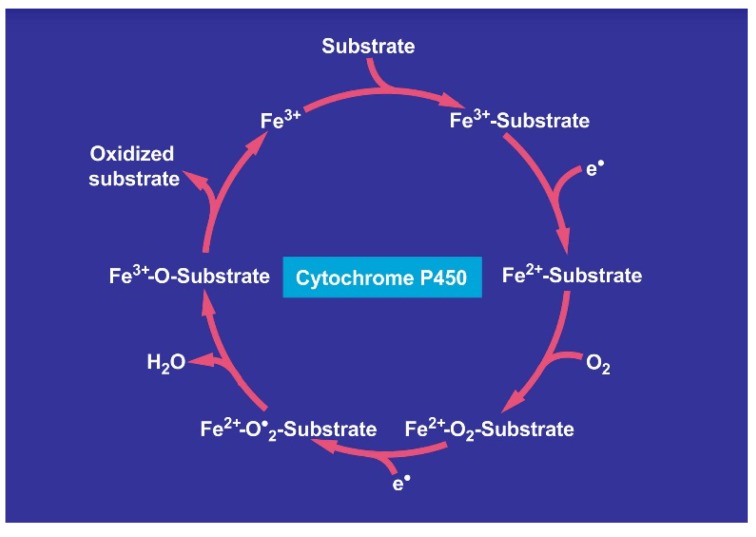
Cycle involving cytochrome P450 for the metabolism of various substrates including carbon tetrachloride.

There is also consensus that hepatic microsomal CYP2E1 is the preferred isoenzyme of CYP responsible for the conversion of CCl_4_ to toxic intermediates [5,6,12,48,49,50,51,52] in analogy to other toxins such as vinyl chloride and dimethylnitrosamine (Figure 19), requiring NADPH-cytochrome 450 reductase, NADPH + H^+^ as reducing equivalent, and O_2_ (Figure 20), with various steps involving CYP using CCl_4_ as substrate and other aliphatic halogenated hydrocarbons (Figure 21). Since experimental administration of CCl_4_ drastically decreased CY2E1, CYP2B, CYP3A2, CYP2C11, and CYP1A2 mRNA and protein expressions [53], CYP isoenzymes others than CYP2E1 may be involved in catalyzing CCl_4_.

#### 6.2.1. Molecular Oxygen

Molecular oxygen is required in the liver cell for the microsomal NADPH-dependent degradation of CCl_4_ (Figure 20 and Figure 21), a process that leads to a variety of intermediates including free radicals and ROS [54]. Referring to anecdotal reports, the suggestion has been made that hyperbaric oxygen treatment may ameliorate CCl_4_ hepatotoxicity in humans and animals [29]. However, the role of oxygen in liver injury by CCl_4_ is still a matter of debate [29,54]. With the CO_2_-induced hyperventilation, pO_2_ values are achieved in the blood that are well within the normal range (Table 6) and are likely achievable in the liver, but it has not been investigated whether this is protective or injurious to the liver. This uncertainty was also discussed for hyperbaric oxygen [29]. Although hyperbaric oxygen treatment with focus on the injury at the microsomal level may be an option for human CCl_4_ intoxications, its superiority over the CO_2_-induced hyperventilation targeting at acceleration of toxin removal has not been established.

#### 6.2.2. Destabilization and Suicidal Inactivation

CCl_4_ causes a rapid decrease in CYP2E1 [55,56]. Mechanism-based inactivation of cytochrome P450 can result in the chemical modification of the heme, the protein, or both as a result of covalent binding of modified heme to the protein. Circumstantial evidence suggests that the inactivation of P4502E1 by CCl_4_ is a result of P4502E1 proteolysis from the microsomal membrane. While there are many potential pathways for protein degradation, the loss of P4502E1 was associated with increased formation of high molecular weight microsomal ubiquitin conjugates. The formation of ubiquitin-conjugated microsomal protein, which correlates with P4502E1 loss, suggests that ubiquitination may represent a proteolytic signal for the rapid and selective proteolysis of certain unstable conformations of P4502E1 from the endoplasmic reticulum [55]. In other studies using quantum chemical calculations, the anaerobic metabolism of CCl_4_ by P450 enzymes was investigated [56]. It was found that the substrate CCl_4_ might undergo one or two subsequent one-electron reductions to generate different reactive metabolites, trichloromethyl radical (˙CCl_3_) and dichlorocarbene (:CCl_2_) respectively. Meanwhile, it was the reduced ferrous heme complex rather than the unreduced ferric heme complex that could directly achieve such reductions. Based on the formation of the former reactive metabolite, a further one-electron reduction could take place with the assistance of a proton to yield the latter reactive species, i.e., a further reductive dechloridation of ˙CCl_3_ could take place. In addition, the ˙CCl_3_ species was capable of binding covalently to the *meso*-carbon atom of the prosthetic group, leading to the suicidal destruction of P450 enzymes. Whereas the :CCl_2_ species with CO as its hydrolysis product was involved in the CCl_4_-dependent reversible P450 inhibition, it was not significantly involved in the CCl_4_-dependent irreversible P450 destruction. It is obvious that the reductive metabolism of CCl_4_ to reactive intermediates by P450 enzymes is an essential prerequisite for its toxicity [56]. Of importance in the clinical context, such destructed CYP isoenzymes are inactive for another injurious metabolic attack by another CCl_4_ molecule. A cascade of events leading to liver injury by CCl_4_ has been described in detail recently [34]. 

#### 6.2.3. Down-Regulation by Glucose

Short-term treatment with 400 g glucose per day is recommended in CCl_4_ intoxication (Table 4) [36] This suggestion is based on the high dosed glucose treatment of acute intermittent porphyria [57] in order to downregulate the hepatic ALA synthase activity, reducing thereby the formation of δ-aminolevulinic acid (ALA) [58,59] and heme as the precursor of the hemoprotein CYP [59]. Indeed, the existence of this ALA-dependent pathway has been verified by studies on incorporation of radioactive-delta-aminolevulinic acid into microsomal CYP [60]. 

#### 6.2.4. Inhibition by Cimetidine

Cimetidine was included as pharmacotherapy in the treatment recommendations for CCl_4_ intoxication (Table 4), based on experimental studies showing that this drug reduced liver injury and lethality in animals with CCl_4_ poisoning [61]. This ameliorating effect is likely due to an inhibitory effect of CYP isoenzymes including CYP2E1 (Figure 20) due to substrate competition of cimetidine versus CCl_4_. Comparative studies are needed to prove whether other injectable drugs or compounds are to be preferred in future cases, but this question is outside the focus of this review. Cimetidine could also inhibit CYP2E1 present in the kidneys [62] possibly responsible for initiating renal injury due to CCl_4_ degradation and leading to renal failure. Therefore, cimetidine may have a dual effect protecting the liver and kidneys in CCl_4_ intoxication. Apart from the therapy using cimetidine, more important is likely providing a priory the patient with sufficient electrolyte infusions combined with furosemide to achieve forced diuresis preventing renal insufficiency by CCl_4_ (Table 4).

#### 6.2.5. Up-Regulation by Alcohol 

CCl_4_ and ethanol share with CYP2E1 a common metabolic pathway (Figure 19, Figure 20 and Figure 21) [5,6] that is induced by chronic alcohol consumption [12]. This induction is viewed as risk factor for liver injury by acute CCl_4_ intoxication in animals, associated with increased covalent binding of ^14^CCl_4_ metabolites to microsomal protein in vitro and an increased metabolism of ^14^CCl_4_ to ^14^CO_2_ [11]. These changes likely occur also in those patients with a history of chronic alcohol consumption prior to the acute CCl_4_ intoxication (Table 1 and Table 7). In animal studies, concomitant acute application of ethanol with CCl_4_ reduces initially the liver injury by CCl_4_ but this is offset later on and replaced by potentiation of liver injury [16]. In other experimental studies, CCl_4_ levels were analyzed under various conditions in animals receiving intragastrically either CCl_4_ alone or combined with ethanol [63]. Three hours after experimental gavage, CCl_4_ levels were higher in the blood, the liver, and fat in the group of animals receiving CCl_4_ combined with ethanol as compared to those treated with CCl_4_ alone. Transferring these results to patients who acutely ingested CCl_4_ simultaneously with ethanol, their risk seems to be increased because they may experience initially higher CCl_4_ levels triggered by ethanol.

Hepatic microsomal cytochrome P450 (CYP), especially its isoenzyme CYP2E1, is also a component of the hepatic microsomal ethanol-oxidizing system (MEOS) involved in the hepatic metabolism of ethanol (Figure 20) [12,13,14]. The CYP2E1 content and MEOS activity are both inducible following prolonged alcohol use [12], whereas for MEOS activity even a single dose of ethanol is sufficient for its induction [15]. It was therefore not unexpected that chronic alcohol consumption predisposes to liver injury by CCl_4_ [11]. Other experimental studies focused on the effect of an acute dose of ethanol on the hepatotoxicity due to a single dose of CCl_4_ [16]. Under clinical aspects, alcohol acutely ingested or used chronically before is a major issue in patients acutely intoxicated by CCl_4_.

On a molecular basis, alcoholic liver injury is due to acetaldehyde (C_2_H_4_O) generated for instance via MEOS from ethanol (C_2_H_5_OH) as its first oxidation product and due to various reactive O_2_-species (ROS) [64]. These include Ethoxy radical CH_3_CH_2_O, Hydroxyethyl radical CH_3_C(**^.^**)HOH, Acetyl radical CH_3_CHO**^.^**, Singlet radical ^1^O_2_, Superoxide radical HO**^.^**_2_, Hydrogen peroxide H_2_O_2_, Hydroxyl radical HO**^•^**, Alkoxyl radical RO**^.^**, and Peroxyl radical ROO**^•^**. Some of these radicals are generated also during CCl_4_ decomposition by CYP 2E1. Because radical formation determines liver injury by ethanol [64] and CCl_4_ [5,6,54], both chemicals follow some type cascade of liver injury events as discussed for CCl_4_ [34] and ethanol [64], a hazardous combination for patients with an alcohol problem who are acutely intoxicated by CCl_4_. 

#### 6.2.6. Preexisting Liver Disease

A crucial question remains as to whether preexisting liver disease may have an impact on acute liver injury by CCl_4_ in humans. Cytochrome P450 isoenzymes 2E1, 2D6, 1A2 and 2C19 contents decline with increasing hepatic disease severity, but their activities were differently affected [65]. For instance, CYP2E1 activity was only lost in patients with decompensated cirrhosis. In the actual cohort with acute CCl_4_ intoxication, decompensated cirrhosis was not diagnosed in any of the patients at admission (Table 7). Therefore, the crucial question raised above remains unresolved regarding severe liver diseases in humans such as decompensated cirrhosis due to lack of clinical evidence. Theoretically, this question can be studied using animal models of prolonged CCl_4_ application to achieve cirrhosis [66,67,68]. Among these, a good approach is the animal model in which both variation and level of critical damage are monitored by the daily weight change of the rat in response to intragastric carbon tetrachloride given during light halothane/oxygen anesthesia; the response each time being used to calibrate the subsequent dose of carbon tetrachloride to fit the individual rat [68]. The method is effective in producing cirrhosis with ascites in about 75% of rats after 8–10 doses of carbon tetrachloride.

In addition, acute liver injury by CCl_4_ will presumably be attenuated by mild pre-existing liver diseases such as nonalcoholic fatty liver disease (NAFLD) or alcoholic fatty liver (AFL) due to increased CCl_4_ metabolism via CYP2E1, the isoenzyme commonly found with increased contents and enzymatic activities in both, NAFLD and AFL [69]. 

## 7. Liver Transplantation

Published data on liver transplantation in patients with acute liver failure due to CCl_4_ poisoning are not available, with the exception of a single patient who was treated by forced ventilation and received an orthotopic liver transplantation [70] but died from aspergillus sepsis after re-transplantation of the liver together with a kidney transplantation. This patient also experienced rhabdomyolysis that has never been described in previous cases. The recommendation is provided to delay transplantation until most of the toxin has been eliminated in order to prevent fatal graft damage. Considering a potential liver transplantation will remain a case by case decision. 

## 8. Summarized Considerations of CCl_4_ Poisoning for Clinical Practice

Due to its highly toxic properties, CCl_4_ has been abandoned as a solvent from the market in many countries or is still used in the industry under strict regulatory surveillance. Nevertheless, acute intoxications by CCl_4_ still occur in humans who incorporated it by ingestion or inhalation causing serious health problems by organ injury including the liver and kidneys. Historical cases are valuable to understand previously limited treatment (Table 1 and Table 2), but progress has been made regarding pathogenesis, diagnosis and treatment of this potentially deleterious CCl_4_ poisoning (Table 3, Table 4, Table 5, Table 6 and Table 7).

It is now clear that the hepatic microsomal CYP 2E1 plays an important pathogenetic role for bioactivation of CCl_4_ to toxic radicals for initiating liver injury, requiring two strategies aiming to early eliminate CCl_4_ by gastro-intestinal lavage and hyperventilation and also to reduce microsomal toxification. Progress has been made in the clinical setting by measurements of CCl_4_ levels in poisoned patients to establish the diagnosis. Care must be taken for various complications that may occur during clinical treatment and have to be diagnosed in time (Table 8). These include disturbances of coagulation, renal injury and failure, respiratory insufficiency, and cardiac arrhythmias. Finally, treatment approaches are now much better defined (Table 4 and Table 5), and the clinical features are clearly described (Table 8). 

In most patients intoxicated by CCl_4_, three phases are clinically apparent whereby the second phase is the interval phase in between with little or no symptoms (Table 8). CCl_4_ intoxication is a serious clinical issue as most centers may not well be prepared treating these patients when admitted, unless the required devices for the CO_2_-induced hyperventilation therapy are available locally and quickly at hands during the patient’s transfer to the center [34] 

## 9. Conclusions

Acute intoxications by CCl_4_ are clinical challenges due to the associated high lethality rate, conditions that require quick initiation of therapy strategies to enhance CCl_4_ elimination via (1) gastro-intestinal lavage to clear the intestinal tract from ingested CCl_4_ and via (2) forced ventilation induced by CO_2_ to accelerate CCl_4_ removal as unchanged chemical via the lungs. As compared to CCl_4_, which in itself is not toxic, its metabolites generated from CCl_4_ via the hepatic microsomal cytochrome P450 2E1 represent toxic radicals that attack cellular structures including proteins and phospholipids leading to apoptosis and cell necrosis. To reduce these toxic events at the microsomal levels, two approaches are beneficial, (1) high doses of glucose should be applied intravenously to downregulate cytochrome P450 levels, and (2) the intravenous application of drugs such as cimetidine with the potency to inhibit cytochrome P450 functions and thereby reducing the conversion of CCl_4_ to toxic radicals. Whereas these two approaches primarily help reduce liver injury, CCl_4_-related renal injury must be circumvented by forced diuresis, keeping in mind that this approach does not help accelerating CCl_4_ removal through the kidneys. It is obvious that various strategies are applicable to reduce the deleterious effects of CCl_4_. 

## Figures and Tables

**Table 1:** Selection of historical cases of liver injury by acute CCl_4_ poisoning.

Year Country	Cases (n)	Case Details	First Author
1922 Ceylon	2	Following ingestion of 13 mL CCl_4_, liver changes were described as granular degeneration of liver cells with leucocytic infiltration in the first patient, and as fatty degeneration of liver cells with diffuse leucocytic infiltration following 24 mL CCl_4_ in the second patient.	Docherty [17]
1932 United States	7	Author reported having observed 7 men with CCl_4_ poisoning in a small plant where it was used as a solvent for cleaning. The use of calcium lactate was discussed, as such pretreatment in animals for 1 to 3 weeks prior to poisoning was found to decrease toxicity.	McGuire [18]
1935 United States	1	Severe case of accidental poisoning with ingested 118–148 mL CCl_4_ mistaken as alcoholic beverage in a man addicted to alcohol. Symptoms included vomiting, severe colicky abdominal pains with numerous watery stools, mental cloudiness and confusion. His family physician treated him with gastric lavage. At hospital treatment included a diet high in carbohydrates (200 g) and daily intravenous administration of dextrose and calcium gluconate, associated with oral administration of 1.8 g of calcium lactate and rectal administration of 10% dextrose (120 mL) every four hours. On day 11 so called sino-auricular tachycardia was documented, and on day 62 sinus arrhythmia. Discharge was after 30 days in good condition.	Lehnherr [19]
1950 United States	12	Histology data are reported from 12 cases of CCl_4_ poisonings, 6 from ingestion and 7 from inhalation. All but one of the 12 patients had an acute alcohol problem or chronic alcoholism. Symptoms included nausea, vomiting, abdominal cramps, malaise, and headaches. Jaundice and renal failure were common. Duration of illness was variable, ranging from 2 to 18 days. There was progressive diminution the necrotic areas in the liver with longer periods of survival. Renal changes focused on the proximal convoluted tubules, described initially as swollen epithelial cells and changing later to a more cloudy swelling with marked granularity of the cytoplasm.	Moon [20]
1955 United States	8 out of 75	A pathology report of 8 patients who died from acute CCl_4_ poisoning, derived from 75 cases of fatal CCl_4_ poisonings on file at the US Armed Forces Institute of Pathology (AFIP). Few patients died from anesthetic effects, most from acute liver or renal failure. Histology findings are detailed described. Liver lesions included confluent zonal necrosis, centrilobular necrosis, and midzonal necrosis. Renal lesions were described as nephrosis, preferentially as fat necrosis. Intoxication was due to inhalation in half of the patients and by ingestion in the other half. Survival after poisoning ranged from 2 to 312 h. Alcohol use was not recorded in one patient, but was associated with CCl_4_ exposure in 3 patients. In the other patients, alcohol use was classified as occasional or moderate in 2 patients and heavy in the remaining two.	Jennings [21]
1958 United States	20	Lethality rate from CCl_4_ intoxication was 25%, with 5/20 cases. Among the 5 patients with fatal outcome, CCl_4_ was inhaled by 3 patients and ingested by 2 patients. Among the initial cohort of 20 patients, 16 had consumed large quantities of alcohol daily for a period of months or years, and 14/16 patients took alcohol shortly before, during or shortly after exposure to CCl_4_. Two patients were not heavy drinkers but had ingested alcohol at the time of CCl_4_ exposure.	Guild [22]
1962 United States	11 out of 19	Report of 19 patients with acute renal failure due to CCl_4_ intoxication, associated with increased serum activities of AST up to 48,000 U/L in 11 patients. Exposure route in the 19 patients was ingestion in 2 patients and inhalation in the other 17 patients. Alcohol ingestion was described in 17/19 patients, vomiting in all 19 patients, diarrhea in 10/19 patients, and abdominal pain in 13/19 patients. Clinical outcome was favorable in 18/19 patients.	New [23]
1966 Israel	1	Patient ingested 20–30 mL CCl_4_ accidentally, was slightly confuse and showed a maximum of serum AST activity of 182 U/L on day 5. His clinical symptoms subsided the next day.	Fischl [24]
1969 Germany	8	CCl_4_ intoxication was by ingestion in 7 patients and by inhalation by one patient. Ingested CCl_4_ ranged from 8 mL to 150 mL. In 7/8 patients renal insuffiency was detected that required dialysis in 6 of these. Most patients had symptoms of gastroenteritis, partially with bleeding. Maximum total bilirubin was 19.5 mg/dL and maximum serum ALT activity was 2396 U/L. Liver histology was unremarkable in in 3 out of 4 patients, whereas in another patient a questionable steatosis and remnants of liver cell necroses were detected 12 weeks after intoxication. Toxic pancreatitis was diagnosed in 3/8 patients. Two patients died from cardiac failure. Overall lethality rate was 2/8 patients equivalent to 25%.	Dume [25]
1972 United Kingdom	1	Intoxication by intentionally ingestion of 120 mL CCl_4_ together with Na-phenobarbital, Carbromal, trichloral, and ethanol. He complained of a burning sensation in his abdomen and throat. Vomiting was not reported, but stomach washout was initiated 2–3 h after ingestion. Maximum serum activities of AST (6700 U/L) and AST (10,700 U/L) were reported on day 4 following ingestion were reported. Maximum serum creatinine (9 mg/dL) was described on day 7, and initially decreased urinary output was successfully treated including also the use of furosemide. Atrial fibrillation commenced on day 4 with a pulse rate of 120–140/min and spontaneous reversal to sinus rhythm on day 22. Hemoglobin fell from 17.5 to 10.2 g/dL. Recovery was complete at discharge on day 29.	Kennaugh [26]
1981 Kuwait	1	Inhalation of CCl_4_ during cleaning machinery on a ship led to hospital admission 10 days after inhalation, when serum activity of AST was 71 U/L and of ALT 90 U/L, with normalization in the further course. Patient experienced myocarditis and required artificial ventilation and dialysis during his 14-day hospital stay.	Hadi [27]
1982 Germany	3	The 3 male patients consumed 2–5 bottles of beer and ingested unintentionally 1–2 swallows (around 20–40 mL) from a bottle containing 20% CCl_4_. Among the symptoms were headaches, vomiting, abdominal pains, and diarrhea in all patients. Dialysis was required in 2/8 patients. Maximum serum ALT activity of was 5500 U/L. Clinical course was otherwise uneventful.	Schäfer [28]
1982 United States	1	Intentional poisoning by ingestion of 250 mL CCl_4_, patient was found in a semicomatose condition and experienced profuse watery diarrhea. Vomiting was not reported. Following 4 h, he was admitted to the hospital. Blood alcohol level was 0.5 mg/dL. A carbon monoxide intoxication was initially suspected due to an erroneously high blood CO level that was later corrected to 4.3 Vol %, and the patient was treated with hyperbaric oxygen at 2.5 atm for 45 min. Hyperbaric oxygen therapy was reinstituted on day 5 and provided until day 16 with 2.0 atm for 2 h twice daily. Maximum serum activities of AST were around 420 U/L on day 5 and of ALT around 700 U/L between days 6 and 7. Additional treatments started 16 h after ingestion and included charcoal, mineral oil laxatives and hypothermia using a cooling blanket. As a diagnostic aid, X-ray of the abdomen revealed radiopaque material in the small bowel and colon, considered to be CCl_4_. Outcome was favorable.	Truss [29]
1983 Canada	1	Patient ingested intentionally 100 mL CCl_4_ and rum, experienced severe liver injury with maximum serum ALT activity of 10,000 U/L on day 2 and renal failure with maximum serum creatinine of 13.8 mg/dL on day 7 requiring hemodialysis. Treatment included also parenteral nutrition. Patient survived.	Fogel [30]
1985 United Kingdom	19	Acute CCl_4_ poisoning in 19 patients with blood CCl_4_ levels ranging from 0.1–31.5 mg/L. Vomiting (11 patients), abdominal pain (5), diarrhea (4), and coma/drowsiness (6) were the most common symptoms and signs. Maximum serum activities of AST was 8070 U/L and of ALT 8600 U/L. Out of 13 patients treated with intravenous *N*-acetylcysteine, 7 patients showed mild hepatic damage, 1 patient had moderate hepatic damage, and 1 patient with a history of alcoholism sustained massive hepatorenal damage and needed hemodialysis. Of the 6 patients (1 lost to follow-up) who were not given *N*-acetylcysteine 3 patients had hepatorenal failure and needed dialysis, and 1 patient died. Prompt treatment with *N*-acetylcysteine was considered to minimize subsequent hepatorenal damage.	Ruprah [31]
1994 Japan	1	Patient with a history of chronic alcohol use ingested CCl_4_ and complained about nausea, vomiting, abdominal pain, and diarrhea. Maximum activity of serum AST was 5160 U/L and of ALT 3000 U/L. Liver histology showed perivenular and centrilobular fibrosis and preferentially in the centrilobular area also liver cell necrosis, ballooned hepatocytes, cellular infiltration and fat droplets. Histology findings likely represent a combination of alcohol use and CCl_4_ ingestion.	Hoshino [32]
2013 Slovakia	60	CCl_4_ intoxication in 60 patients, lethality rate of 3.3%. No case details were provided in the report.	Mydlík [33]

Abbreviations: ALT, alanine transaminase; AST, aspartate transaminase.

**Table 2:** Summary of historical data on CCl_4_ intoxication.

Points of Clinical Interest
Starting from 1922, data from historical reports in humans provide the following facts: ● The historical use of CCl_4_ as anthelmintic chemical is obsolete and was early recognized ● CCl_4_ is hepatotoxic and nephrotoxic, toxicities may occur one after the other, together, or alone in a patient, but risk factors that determine the sequence of injurious events are unknown ● Most poisonings occur by ingestion or inhalation ●The degree of cellular toxicity in liver and kidney is independent from the route of toxin uptake ● A close dose dependency of liver injury by CCl_4_ is not apparent in the reported inhomogeneous cohorts ● A lethal dose of CCl_4_ cannot be established with certainty due to many confounding variables of toxin uptake ● Vomiting is a frequent early symptom after CCl_4_ inhalation or ingestion, facilitating toxin removal ● Profuse diarrhea helps accelerating toxin removal after CCl_4_ ingestion ● Alcohol use is commonly described, preexisting as chronic abuse or acutely at occasion of CCl_4_ poisoning ● Several methods for primary toxin removal from the gastro-intestinal tract have been used, including gastric lavage, using charcoal or paraffin, but sufficient evidence of efficacy was not provided ● Under the assumption of potential benefit, several compounds have been administered, such as iron ● Little new evidence was provided of attempting to remove more effectively the toxin ● Effective liver transplantation in acute liver failure by CCl_4_ has not been reported ● Patients who survived an acute CCl_4_ intoxication commonly have a good prognosis without permanent injury but results of stringent follow-up studies were not published ● Lethality rates of acute CCl_4_ intoxication were found in a broad range from 2% up to 25% ● Specific causes of death were rarely reported but most were likely due to hepatic or renal injury ● No attempt was made to introduce drugs for inhibition the microsomal metabolism of CCl_4_ to toxic metabolites ● Liquemin use was not addressed ● Clinical cohorts lacking homogeneity ● A biphasic clinical course with a free interval was early recognized ● X-ray abdomen to detect possible radiopaque material of the small bowel and colon as sign of suspected CCl_4_ ● Early descriptions of cardiac arrhythmias, anemia, and anuria

**Table 3:** Diagnosis of liver injury by acute CCl_4_ intoxication.

Diagnostic Criteria
1. History of poison administration 2. Details on route of CCl_4_ uptake 3. Assessing uptake of CCl_4_ amount and vomiting to estimate uptake of total dose 4. Initial symptoms may rarely include narcosis 5. Assessment of alcohol use prior to intoxication and at time of poisoning to estimate extra risk of ethanol for liver injury by CCl_4_ 6. X-ray abdomen that may show opaque changes in the intestinal tract suggestive of intraluminal CCl_4_. 7. Qualitative and semi-quantitative analysis of CCl_4_ in the exhalation air using the Draeger-tube^®^ system (DTS) 8. Quantitative analysis of CCl_4_ in the blood using the heads-space technique of gas chromatography 9. Routine laboratory evaluation as commonly done in a setting of an intensive care unit 10. Special care is needed to early recognize respiratory insufficiency, alcohol withdrawal symptoms, renal insufficiency acute renal failure, liver function disturbances and acute liver failure, cardiac arrhythmias, anemia, toxic bone marrow injury, disseminated intravascular coagulation (DIC), blood glucose in face of high glucose administration.

For toxin detection in the exhalation air, the use of the Draeger-tube^®^ system (DTS) supplied by Draeger, Lübeck in Germany, is recommended [34,35,36]. According to the information of the manufacturer, DrägerTubes^®^ are glass vials filled with a chemical reagent that reacts to a specific chemical or family of chemicals [35]. A calibrated 100 mL sample of air is drawn through the tube with the Dräger accuro^®^ bellows pump. If the targeted chemical is present, the reagent in the tube changes color, and the length of the color change typically indicates the measured concentration.

**Table 4:** Therapy of adult patients with acute CCl_4_ intoxication.

Therapy Approaches	Ingestion	Inhalation
1. Endotracheal intubation prior to intended gastro-intestinal lavage after evaluation for risk of aspiration	+	-
2. Primary toxin elimination by gastro-intestinal lavage in the intubated patient. Endoscopic removal of the ingested hydrocarbon from the stomach is not recommended, because the toxin by virtue of its strong solvent property may damage parts of the gastroscope. For activated charcoal and paraffin, no evidence for clinical efficacy in CCl_4_ poisoning exists	+	-
3. Forced ventilation by CO_2_-induced hyperventilation therapy aims to accelerate toxin removal by exhalation and should be maintained until abnormal laboratory tests such as liver and kidney parameters approach normal values	+	+
4. Central venous access	+	+
5. Intravenous cimetidine as bolus (200 mg) for inhibition of CCl_4_ degradation by CYP, then 1600 mg for the initial 24 h via infusion pump and for the subsequent days	+	+
6. Intravenous 400 g glucose/24 h and on subsequent days to down-regulate CYP to reduce CCl_4_ degradation	+	+
7. Intravenous electrolytes plus furosemide aiming forced diuresis to prevent renal failure	+	+
8. Liquemin 15,000 IU/24 h and on subsequent days to minimize the risk disseminated intravascular coagulation (DIC)	+	+

Adapted and updated from a previous report [34]. Abbreviation: CYP, Cytochrome P450.

**Table 5:** CO_2_-induced hyperventilation therapy for acute CCl_4_ intoxication.

CO_2_-Induced Hyperventilation in Adults
**Aim of therapy**
Forced CO_2_-induced hyperventilation is also called CO_2_-induced hyperventilation therapy to specifically indicate its therapy goal. It aims to increase pulmonary excretion of CCl_4_, ideally in an adult or adolescent patient lacking respiratory insufficiency.
**Sufficient spontaneous respiration**
Therapy should be started right after gastro-intestinal lavage has been completed and when endotracheal intubation is not any more needed. Forced hyperventilation is inaugurated by CO_2,_ applied to the patient in a sitting bed position. CO_2_ must be of pure quality and suitable for human use, commonly supplied in gas cylinders, safely placed nearby the bed and the head of the patient. The patient inhales the CO_2_ after passing through a humidifier, a sealed tube, known as nasal oxygen tube. Concomitantly, the patient inspires usual air by open mouth and alternately expires the contaminated air by mouth. CO_2_ is applied at a flow rate of 2–3 L per minute, and with regular inspiration via the nasal tube, an increased respiratory minute volume of up to 25–30 L should be achieved [8]. If the nasal tube is not tolerated, it can be replaced by CO_2_ application through a common, not tightly placed oxygen mask, but this approach is less effective and associated with the risk of again inhaling the contaminated exhalation mixture. Alternatively, a modern viable oxygen mask may perhaps be used with valves inside of these tight-fitting masks may be used that control the flow of gases into and out of the masks, so that rebreathing of exhaled gas is minimized. However, experience with such modern masks is not yet available in the context of CO_2_-induced hyperventilation.
**Insufficient spontaneous respiration**
Patients with respiratory insufficiency require endotracheal intubation for artificial respiration device, using for instance a Bird-system; CO_2_ at a flow rate of 2–3 L per minute is then added to the respiratory mixture to achieve hyperventilation. This is a critical clinical situation for which decisions are required on a case by case basis. In general, patients with a preexisting chronic obstructive pulmonary disease should not be candidates for a CO_2_-induced hyperventilation therapy due to the unfavorable benefit versus risk constellation. As this therapy is not without risks, skilled physicians preferentially pulmonologists should take care for these patients in a setting of an intensive care unit. The therapy requires a 24 h surveillance of the patient with regular measurements of the respiratory minute volume as well as blood gas analyses in order to early recognize complications. Needless to say, constant room ventilation is required to remove the exhaled toxin.

As treatment conditions differ substantially from those in children [9], separate recommendations are given here for the hyperventilation therapy in adults with acute CCl_4_ intoxication as applied in actual cases of Table 7. Adapted from a previous report [34].

**Table 6:** Acid-base balance under CO_2_-induced hyperventilation.

Patients	pO_2_ (mmHg)	pCO_2_ (mmHg)	pH	CHO_3_^−^ (mval/L)
Normal range	81–99	25–45	7.36–7.44	22–26
Patient 1	100 ± 11	49 ± 1	7.37 ± 0.01	26 ± 2
Patient 2	103 ± 4	45 ± 3	7.41 ± 0.01	27 ± 1
Patient 3	95 ± 2	50 ± 3	7.33 ± 0.03	23 ± 1
Patient 4	84 ± 16	42 ± 3	7.47 ± 0.02	29 ± 1
Patient 5	87 ± 5	41 ± 2	7.40 ± 0.02	24 ± 1

Analysis of acid base balance under CO_2_-induced hyperventilation therapy in 5 patients with acute intoxication by ingested CCl_4_, from a previous report [41]. Hyperventilation was achieved using CO_2_ with a flow rate of 2–3 L min^−1^, added to the inspiration air and applied via a nose tube to achieve a respiratory volume of 30 L min^−1^.

**Table 7:** Clinical details of 16 patients with acute CCl_4_ intoxication treated with the CO_2_-induced hyperventilation.

Case	Intoxication	Case Details
1. Male 15 years	Carbon tetrachloride Ingestion (30 mL)	Patient swallowed intentionally 30 mL CCl_4_ and experienced twice vomiting before he was treated in a regional hospital by gastro-intestinal lavage. At admission in our intensive care unit 9 h after intoxication, he was mentally conscious and CCl_4_ was detected in the expiration air presently and during the next 4 days, as analyzed by the Draeger-tube^®^ system (DTS). CO_2_-induced hyperventilation was started and continued for 11 days. CO_2_ was applied via a nasal tube at a flow rate of 2–4 L/min and resulted in a respiratory volume of up to 30 L/min. On day 3 after intoxication, Serum activities of liver enzymes increased and reached a maximum on day 4 (AST 59 U/L, ALT 56 U/L, GDH 18 U/L) and normalized during the next days until day 14 after intoxication. Liver biopsy on day 13 showed no abnormalities. Patient was discharged on day 15.
2. Female 14 years	Carbon tetrachloride Ingestion (10–20 mL)	Patient ingested intentionally 10–20 mL CCl_4_ accessed to from a dry cleaning business where her mother was employed. She was initially admitted to a regional hospital 1.5 h after ingestion where a solvent exhalation smell was realized of the fully oriented patient. A gastro-intestinal lavage was initiated and paraffin was given before she was transferred to our intensive care unit using an emergency car with a doctor. During the transfer she vomited several times and wet herself. At admission 7 h after ingestion, CCl_4_ was 10 and 20 ppm as analyzed by DTS, she was fully oriented and received a CO_2_-induced hyperventilation therapy for 4 days. CO_2_ was given via a nose tube at a flow rate between 2 and 5 L/min, which resulted in a respiratory rate ranging from 24/min to 42/min, associated with a respiratory volume between 21.5 and 33.2 L/min. arterial PO_2_ was in a range of 94–98%, arterial pH was between 7.26 and 7.39, and CHO_3_^−^ between 22.1 and 25.5 mval/L. ECG was always unremarkable. During treatment, creatinine and total bilirubin were always normal, INR was 1.5. At admission, liver tests were within the normal range and marginally increased on day 4 for AST (42 U/L) and ALT (33 U/L). Liver histology by hematoxylin and eosin staining 4 days after termination of the CO_2_-induced hyperventilation therapy showed no overt liver cell necrosis but severe micro-vesicular fatty liver in 80–90% of the liver cells. With a body weight of 48.6 kg, a height of 1.67 m, and a resulting BMI of 17.4 kg/m^2^, pre-existing nonalcoholic fatty liver disease was unlikely. By electron microscopy, cristae of mitochondria are reduced and disorganized. Within the mitochondria crystalline inclusion bodies are found. A few mitochondria represent mega-mitochondria. Abundant lysosomes are found. Discharge was 10 days after ingestion, all laboratory values were in the normal range at that time.
3. Male 31 years	Carbon tetrachloride Ingestion (50 mL)	Patient intentionally swallowed 50 mL CCl_4_ and was found by his mother. After initial treatment in a local hospital and recurrent vomiting, he was admitted at our intensive care unit the same day, where he received a gastro-intestinal lavage and was started on the usual CO_2_-induced hyperventilation therapy for 6 days, achieved with CO_2_ 3–5 L/min. CCl_4_ was detected in the expiration air using the DTS and confirmed in the blood by GC. Clinical course was uneventful, except for a short increase of AST (66–70 U/L) and ALT (123–170 U/L) during day 3 to 5 after ingestion. Patient was discharged 3 days after termination of the hyperventilation.
4. Male 70 years	Carbon tetrachloride Ingestion (~50 mL)	Patient ingested unintentionally ~50 mL CCl_4_ contained in a bottle labelled erroneously as lemonade. Within 30 min thereafter, he experienced diarrhea with black-colored stools, later also recurrent vomiting. At admission in a local hospital he was sleepy and was transferred the same day to our intensive care unit for gastro-intestinal lavage and CO_2_-induduced hyperventilation for 8 days. Presence of CCl_4_ in the blood was confirmed by GC at several occasions. Peak serum activities for AST (92 U/L) and ALT (215 U/L) were observed on day 7 after ingestion. After termination of the hyperventilation therapy, the patient was re-transferred to his local hospital.
5. Male 40 years	Carbon tetrachloride Ingestion (100 mL)	Patient intentionally swallowed 100 mL CCl_4_, was initially treated in a local hospital with gastrointestinal lavage and endotracheal intubation for initiating CO_2_-induced hyperventilation also during transport via plane to our intensive care unit at the same day. At arrival, CCl_4_ was detected in the expiration air using the DTS, and after extubation the hyperventilation therapy was continued with CO_2_ up to 4 L/min via nasal tube for overall 10 days, resulting in a minute respiration rate of 25–30 L/min. The patient was somnolent during several days, an unusual clinical observation. Maximum values for serum activities of AST (5735 U/L) and ALT (3821 U/L) were observed on day 4 after ingestion, which was associated with a maximum increase of serum creatinine (2.1 mg/dL). Liver histology by hematoxylin and eosin stain on day 14 after ingestion revealed moderate centrilobular micro-vesicular fatty liver with few liver cell necrosis. Electron microscopy with 5200-fold magnification showed a striking proliferation and pronounced dilatation of the smooth endoplasmic reticulum of the hepatocyte, presenting as dilated cisterns. Few mitochondria are enlarged and most mitochondria are injured. Abnormal laboratory results returned to normal values rapidly, and the patient was discharged 14 days after ingestion.
6. Male 16 years	Carbon tetrachloride Ingestion (~30 mL)	Patient ingested intentionally CCl_4_ (~30 mL) and was admitted to a local hospital for gastro-intestinal lavage. Documented are recurrent vomiting, nausea and dizziness. On the day of ingestion, the fully oriented patient was transferred to our intensive care unit for CO_2_-induced hyperventilation, done for 10 days with CO_2_ (3–5 L/min), which resulted in a respiratory frequency of 28–36/min and a respiratory minute volume of 21–28 L/min. On day 5 after ingestion, peak values were determined for AST (59 U/L) and for ALT (56 U/L), which rapidly normalized within few days. Liver histology 13 days after ingestion showed minimum steatosis in a few liver cells and single liver cell necrosis. Discharge was on day 14 after ingestion.
7. Female 50 years	Carbon tetrachloride Inhalation (~ 10 mL)	Patient inhaled unintentionally CCl_4_ (~10 mL), which she used for cleaning of a spot on her carpet. She experienced nausea that persisted for 1 day until she approached a local hospital, which arranged the transfer to our intensive care unit for initiating CO_2_-induced hyperventilation therapy, which was accomplished in the usual way by nasal tube. Here CCl_4_ was not detectable in the expiration air through the DTS, but in the blood ethanol was detected with 1.77‰. The hyperventilation therapy was applied for 3 days, Initial serum activities of AST (16 U/L) and ALT (20 U/L) remained virtually unchanged during the following days, ranging from 8 to 20 U/L. Discharge was on day 4 after intoxication.
8. Female 50 years	Carbon tetrachloride Ingestion (~50 mL)	Patient unintentionally took one swallow of CCl_4_ contained in a mineral water bottle, vomited intentionally and drank 0.5 L milk thereafter. During the following hours at home, she suffered from severe headaches and nausea before she arranged admission to a local hospital the next day. After transfer to our intensive care unit, CCl_4_ was detected in the expiration air by the DTS, and the patient received the usual CO_2_-inducded hyperventilation therapy, requiring CO_2_ (2–3 L/min) to achieve a respiratory frequency of 24–30/min. After 5 days, the therapy was terminated. Peak enzyme activities were determined for serum AST (1600 U/L) and ALT (2560 U/L) 4 days after ingestion, with retarded decline and normalization during the following days. Liver biopsy was not done. Discharge from our hospital was 9 days after ingestion.
9. Male 36 years	Carbon tetrachloride Ingestion (50 mL)	Patient intentionally ingested 50 mL CCl_4_. At the same time, he consumed beer (~2.5 L) and hard liquor (~0.5 L), vomiting in the further course was negated. For many years before, he drank 1–2 L beer daily but no hard liquors. At admission to the local hospital 5 h after CCl_4_ ingestion, his blood ethanol was 2.5–3.0‰. Gastro-intestinal lavage was initiated and transfer to our intensive care unit was organized where he arrived 6.5 h after CCl_4_ ingestion. At admission, presence of CCl_4_ in the blood was confirmed by head-space GC. Serum liver tests were all in the normal range: AST (10 U/L), ALT (14 U/L), and GGT (9 U/L), associated with marginally increased serum total bilirubin (1.5 mg/dL) and normal serum creatinine (0.9 mg/dL). Hyperventilation was initiated with CO_2_, initially with 2 L/min and intermittently with up to 4 L/min via nasal tube. Respiratory volume commonly was 15–33 L/min and respiratory frequency 15–28/min. On day 3 after CCl_4_ ingestion, a peak was observed for AST (8960 U/L) and AST (4200 U/L), with a subsequent decline. Total bilirubin was elevated with 1.5 mg/dL on the day after ingestion and increased steadily up to 18.9 mg/dL (with 15.8 mg/dL direct bilirubin) on day 24, followed by subsequent decline. Serum creatinine started with 1.4 mg/dL on day 2 after ingestion to increase and reached values of up to 19.7 mg/dL on day 35. Endotracheal intubation was required on day 6, and dialysis was started on day 18 following CCl_4_ ingestion. The further clinical course was complicated by 2 reanimations, acute liver failure and a pneumonia that developed on day 34 following CCl_4_ ingestion and was not treatable with antibiotics. Along the treatment, blood CCl_4_ levels as determined by head space GC were high at beginning, and much lower in the further course. The cause of death was classified as respiratory insufficiency along with multi-organ failure, related to acute CCl_4_ intoxication with excessive alcohol use as risk factor.
10. Female 22 years	Carbon tetrachloride Inhalation (~50 mL)	Patient inhaled intentionally CCl_4_ (~50 mL) and remained at home. Next day she noticed some nausea, and the other day she experienced increasing symptoms of nausea and stomach cramps. Following presentation at a local hospital she was transferred to our intensive care unit for CO_2_-induced hyperventilation. At admission, she was alert and had tachycardia with a pulse rate of 120/min but in the further course she was intermittently somnolent. By head space GC CCl_4_ was not detectable in the blood. CO_2_-induced hyperventilation via nose tube was initiated and performed for 11 days. Maximum increases were found for serum activities of AST (6770 U/L) and ALT (7990 U/L), but subsequent decline was prompt. At discharge on day 11 after intoxication she was in good condition, AST was 43 U/L and ALT 271 U/L.
11. Male 33 years	Carbon tetrachloride Inhalation (unknown amount)	Patient unintentionally inhaled CCl_4_ for several days_,_ classified as occupational intoxication. As a conservator and owner of a business for restoring oil paintings and art work made of wood, he used for the past 15 years several solvents in small amounts, including trichloroethylene and benzene but started recently using CCl_4_ for 4 days. When he first worked with CCl_4_ to remove wax from oil paintings, he felt sick on days 3 and 4, was subfebrile (38.5 °C axillar), and suffered from loss of appetite, nausea, vomiting, headaches and pains in the neck. He also noticed a brown urine color like German dark beer but could not remember the color of his stool. He then closed his business during the following Christmas season. The day after closing, his condition improved substantially, and after two more days outside of his working place he recovered completely and was well for the next days. After new year, he resumed working with CCl_4_, and after 2 days he suffered again from nausea, headaches, and colored urine. His family physician organized his admission in a university hospital, from which the patient was transferred to our intensive care unit. At the day of admission, CCl_4_ was detected with 2 ppm in the expiration air by the DTS and CO_2_-induced hyperventilation therapy was initiated and provided for 4 days using the nasal tube approach. CO_2_ was given at 1.8–2.0 L/min that led to a respiratory volume of 36 L/min. Throughout the clinical course, values of serum total bilirubin and creatinine remained in the normal range. Previous alcohol use of 0.7 L wine and 0.2 L beer daily was considered as risk factor of the liver toxicity by CCl_4_. On day 5 after admission, liver histology showed a moderate steatosis with small and large fat droplets as well as some inflammation but no necrosis. By electron microscopy, the mitochondria are slightly swollen and their cristae are reduced. Abundant bile pigments were seen between nucleus and a bile canaliculus. Initial laboratory analyses revealed increased serum activities for AST (1277 U/L) and ALT (1177 U/L) with a continuous fall during the next days. At discharge after a 7 day stay in the hospital, AST was normal with 21 U/L and ALT moderately increased with 201 U/L. The initially increased serum GGT of 138 U/L was likely due to prior alcohol abuse, GGT at discharge was 124 U/L.
12. Female 29 years	Carbon tetrachloride Inhalation (unknown amount)	Patient inhaled unintentionally CCl_4_ under similar working conditions as described by patient 10 above, in whose business she was employed as conservator. For 4–5 weeks she was busy removing a wax surface from several oil paintings, which was facilitated when she used a solvent containing CCl_4_ derived from a 3 container. She worked under conditions of an open window in a distance of around 7 m from the deposited, solvent containing oil paintings, but noticed solvent containing vapors, which she obviously inhaled, not considering that CCl_4_ is heavier than air and undulates just above the floor making an open window inefficient. During this work, she experienced malaise, loss of appetite, ever, dark urine, flu-like joint pains and back pains. Others who worked with her in the same room reported on similar complaints. With increasing severity of her symptoms she discontinued working, and physicians of a university hospital suspected an intoxication by CCl_4_ and arranged a helicopter flight for further treatment by forced ventilation to our intensive care unit, where CCl_4_ could not be detected in the expiration air using the DTS, and CO_2_-induced hyperventilation was initiated and carried on for 8 days, using CO_2_ at 2.5–3.0 L/min. Her alcohol use was quantified as 2 L wine per week, her serum GGT was initially 102 U/L and then fluctuated between 109 and 233 U/L. Liver histology obtained 3 days after cessation of the hyperventilation therapy showed in zone 3: a mild steatosis involving 20–30% of the hepatocytes, severe single cell necroses, and a moderate activation of hepatic stellate cells. The clinical course was complicated by oliguric renal insufficiency with creatinine values up to 6.5 mg/dL, treated with forced diuresis. With a normal hemoglobin of 13.2 g/dL initially, remarkable was an emerging anemia with a reduced hemoglobin of 8.7 g/dL of unknown etiology. Total bilirubin undulated between 2.2 and 3.3 mg/dL, and serum GDH activity was initially 1534 U/L and declined quickly. The initially increased serum activities of AST (2545 U/L) and ALT (2645 U/L) normalized until day 11 before she was discharged on day 14. This case as well as the 2 cases above had been reported to the respective trade association.
13. Male 31 years	Carbon tetrachloride Inhalation (unknown amount)	Patient unintentionally inhaled CCl_4_ and had worked together with patient 11 above in the business of patient 10 above under similar working conditions. He reported that the solvents were commonly used outdoors and rarely indoors, except when temperature is low outdoors as in winter. He actually worked indoors in a closed room on 2 days apart from each other with CCl_4_ taken from a 3-L bottle of this solvent. On the first working day he used CCl_4_ for 1 h and 3 days later for 4 h. On day 4, he experienced nausea, headaches, joint pains, lower back pains, sore throat, dark urine, vomiting and diarrhea. Via a university hospital he was transferred with helicopter together with patient 11 to our intensive care unit for treatment. At admission, CCl_4_ was not detected in the expiration air by DTS. He showed beginning withdrawal symptoms which were decreasing in severity during the further course without specific drug treatment and were likely related to his alcohol use reported as 2.5 L beer daily and occasionally more. Hyperventilation was initiated and induced by CO_2_ (2–3 L/min) by nasal tube to reach an expiration volume of 25–20/min. Not well tolerated by the patient, this therapy was ceased on day 5, at that time CCl_4_ was not detected in the blood using the head-space GC technique. At admission, serum GDH activity was 4746 U/L and normalized within 10 days, whereas serum activities were increased for AST (6475 U/L) and for ALT (2143 U/L) and normalized until day 17. Throughout the clinical course, total bilirubin and GGT remained in the normal range. Liver histology obtained 39 days after last CCl_4_ exposure and 40 days after admission showed a low graded fatty liver and residues of a toxic event. The clinical course was complicated by respiratory insufficiency requiring O_2_ application, and renal insufficiency with serum creatinine values that ranged initially from 1.5 mg/dL to 5.3 mg/dL but increased to 14.5 mg/dL on day 5 requiring intermittent hemodialysis on 12 days. An incipient pneumonia in the basal parts of the left lung was successfully treated with ampicillin. Due to these complications, the hospital stay was prolonged, and discharge was possible after 42 days in fairly good condition. This case as well as the 2 cases above had been reported to the respective trade association.
14. Male 21 years	Carbon tetrachloride Ingestion together with Diethyl ether (unknown amounts)	Patient swallowed intentionally CCl_4_ and Diethyl ether, both were used in unknown amounts and verified in the expiration air by DTS. In addition, he ingested ~30 mL Clenbuterol, a decongestant, and Oxeladin, a cough syrup, in an unknown amount. After short-term narcosis, CO_2_-induced hyperventilation was initiated and provided for 7 days. On day 4, serum activities of AST (65 U/L) and ALT (49 U/L) were minimally elevated but otherwise remained unchanged. Discharge was on day 5.
15. Male 16 years	Carbon tetrachloride Inhalation together with Trichloroethylene, Tetrachloroethylene, Diethyl ether (unknown amounts)	Patient inhaled intentionally several solvents from a cloth that he soaked before. He was found unconscious by his parents and woke up 2 h after he was found. He received CO_2_-induced hyperventilation during the transport to our intensive care unit, where all solvents were confirmed in the expiration air using the DTS and hyperventilation therapy was continued for 5 more days. Serum activities of AST and ALT were normal at admission and remained unchanged during subsequent treatment except on day 4 (AST 60 U/L, ALT 53 U/L). He was discharged on day 6 after admission.
16. Female 41 years	Carbon tetrachloride Inhalation together with Tetrachloroethylene (unknown amounts)	Patient inhaled intentionally carbon tetrachloride of unknown amounts together with several other solvents again without clearly documented amounts. Presence of all inhaled solvents was ascertained in the expiration air using DTS. She experienced narcosis for 30 min after she was found and received CO_2_-induced hyperventilation therapy by nasal tube for 48.5 h. Maximum serum activity of AST was 51 U/L and of ALT 48 U/L. Her clinical course was uneventful. Discharge from the hospital was on day 5.

**Table 8:** Clinical characteristics of CCl_4_ liver injury.

Clinical Details of CCl_4_ Liver Injury
● The use of CCl_4_ as a solvent is dangerous due to the risk of liver and kidney injury with potential fatal outcome. ● CCl_4_ poisonings occur even if the solvent is used in small amounts such as for cleaning a carpet in an apartment. ● Commercial use of CCl_4_ is unlawful and dangerous even in a room with open windows and erroneously assumed sufficient ventilation since CCl_4_ is heavier than room air and undulates above the floor to be easily inhaled when working nearby the floor. ● Triphasic course with free interval. ● Phase 1 (often) Beginning with headaches and gastrointestinal symptoms such as vomiting, abdominal or colicky pains diarrhea ● Phase 2 (common) Free interval without symptoms, however with increasing liver tests without causing symptoms ● Phase 3 (rare) Overt liver disease, liver failure, renal insufficiency respiratory insufficiency cardiac arrhythmias ● Qualitative and quantitative analysis of CCl_4_ as the suspected toxin, using GC for analysis in the blood and DTS for analysis in the expiration air. ● Complications emerging due to the intoxication or alcohol abstinence including withdrawal symptoms must early be recognized and treated. ● The hyperventilation therapy is not without risks, especially in patients who require endotracheal intubation. A careful observation of all patients and technical analyses are mandatory for risk minimizing. ● An absolute alcohol abstinence is recommended for 3 months after discharge, because even if the serum activities of the aminotransferases returned to normal values, liver histology obtained at around discharge still showed liver cell necroses by light microscopy, and electron microscopy assessment commonly showed severe hepatic mitochondrial injury. Therefore, a second hit by alcohol must be prevented. ● Long-term hepatic sequelae, such as vanishing bile duct syndrome, have not been reported for a few assessable patients who experienced acute CCl_4_ intoxications.

Abbreviations: DTS, Draeger-tube^®^ system; GC, Gas chromatography. Adapted from a previous report [34].
